# Feature Ranking on Small Samples: A Bayes-Based Approach

**DOI:** 10.3390/e27080773

**Published:** 2025-07-22

**Authors:** Aleksandra Vatian, Natalia Gusarova, Ivan Tomilov

**Affiliations:** School of Translational Information Technologies, ITMO University, 197101 St. Petersburg, Russia; nfgusarova@itmo.ru (N.G.); ivtomilov@itmo.ru (I.T.)

**Keywords:** feature ranking, small samples, Bayesian approach, ranking algorithm benchmarking

## Abstract

In the modern world, there is a need to provide a better understanding of the importance or relevance of the available descriptive features for predicting target attributes to solve the feature ranking problem. Among the published works, the vast majority are devoted to the problems of feature selection and extraction, and not the problems of their ranking. In this paper, we propose a novel method based on the Bayesian approach that allows us to not only to build a methodically justified way of ranking features on small datasets, but also to methodically solve the problem of benchmarking the results obtained by various ranking algorithms. The proposed method is also model-free, since no restrictions are imposed on the model. We carry out an experimental comparison of our proposed method with the classical frequency method. For this, we use two synthetic datasets and two public medical datasets. As a result, we show that the proposed ranking method has a high level of self-consistency (stability) already at the level of 50 samples, which is greatly improved compared to classical logistic regression and SHAP ranking. All the experiments performed confirm our theoretical conclusions: with the growth of the sample, an increasing trend of mutual consistency is observed, and our method demonstrates at least comparable results, and often results superior to other methods in the values of self-consistency and monotonicity. The proposed method can be applied to a wide class of rankings of influence factors on small samples, including industrial tasks, forensics, psychology, etc.

## 1. Introduction

Today, people have to act in an uncertain environment and cannot be fully aware of each situation, let alone control it. Therefore, they need predictive modeling for a better assessment of the available descriptive attributes to predict target attributes, that is, to solve the feature ranking problem [1]. The feature ranking (FR) algorithm forms a list (also called feature ranking) of descriptive attributes ordered by their importance (relevance) in terms of the target attribute(s).

Although the FR problem has been reported for decades [2], it has been constantly expanding to new areas, including geological exploration [3,4], assessing safety risks and staff well-being in industry [5,6], organizing supply chains [7], protecting the network infrastructure of an enterprise [8,9], compensating for environmental damage caused by a particular enterprise [10,11], and the management of patients with comorbidities [12]. Here, it is the small size of the available dataset that becomes a critical limitation.

The FR task implies an adequate choice of the most effective statistical evaluation algorithm. Various criteria have been suggested for this choice, commonly related to the filter, embedding, and wrapper approaches.

Filter methods are data-driven; that is, the criteria for ranking attributes are based on the properties of the data selected by some external axiomatics or heuristics rather than on the results of a model. Filter methods would normally be applicable to large datasets. In particular, ANOVA, the Mann–Whitney test, the R2 test, and similar methods of descriptive statistics are widely used in medical statistics [13,14,15]. In practice, the well-known trade-off between the stability and the accuracy of results in medical applications of descriptive statistics is generally resolved in favor of accuracy. This stipulates using only large samples (cohorts) of patients for analysis. For example, Ref. [15] used a sample of 78,000 patients to rank features. The methods of the Feature Screening group [16] use heuristics aimed at processing ultra-high-dimensional datasets, which is beyond the scope of our work. Thus, Deep Feature Screening [17] uses a neural network for this processing. In terms of processing small samples, the method based on Vendi Score Importance [18] is worth discussing. It groups features by their level of influence on the target class, with subsequent selection of the most effective features. However, as the dataset size decreases, the grouping may become less informative. In addition, with a larger set of features, this method may experience “the curse of dimensionality” and result in insufficient and unstable separation of features by importance. The method described in [19] uses a permutation test, one of the few types of tests in classical statistics that can be adequately applied to small samples. On the other hand, the above tests can be overly conservative; that is, they might be biased towards the irrelevance of features.

The construction of filter methods also uses indicators based on information theory [20,21,22], including Information Gain (IG) [23], mutual information (MI) [24], and the Maximal Information Coefficient (MIC) [25]. To some extent, they exploit the idea of estimating the difference between the probability distributions *p* of features *x* = {*x_i_*}, *i* = 1, …*d*, for different classes *x* = {*y_k_*}, *k* = 1, …*l*, requiring no training of classifiers. However, the distribution is estimated directly from the labeled training dataset, which fundamentally reduces their applicability on small datasets. In addition, this estimate is indirect, that is, the calculated value *p*(*x*|*y*) indicates the contribution of a feature to a particular class. For example, in the MRMR method [24], which is one of the filter methods based on MI, the mutual information is estimated through a Parzen window, which can become suboptimal on small samples. Additionally, feature selection is performed using a greedy algorithm, which can also lead to suboptimal feature selection. As experimentally shown in [20], the results may still tend to degrade with a decreasing sample size, although this is less pronounced than in descriptive statistics.

Embedded methods are also a common choice for FR. They are based on the parameters or structure of regressions, decision trees, SVMs (Support Vector Machines), and other algorithms, providing an easily interpretable assessment of the variables’ importances. For example, in the case of logistic regression, *y* = σ(*w*⋅*x* + *b*), the “organic” measure of the importance of feature *f_i_* is the absolute value of its weight coefficient, (*f_i_*) = |*w_i_*|. Due to this methodological transparency, embedded methods are widely used in applied medical solutions [26,27,28,29,30]. These methods appear to be simple; however, most often they are based on classical algorithms that may have problems with overfitting on small samples.

The effectiveness of embedded feature ranking methods depends on how adequately the models themselves work with small samples. For example, the Lasso [31] and ElasticNet [32] methods use regularization, increasing convergence on small samples. On the other hand, since only regression coefficients are used as a ranking feature, these methods do not appear to properly account for the relationships between variables. Tree-based models, such as Extreme Gradient Boosting (XGBoost) [33] or Random Forest [34], form a sample estimate of the data distribution in the subspace based on the frequency of using individual features, and the estimate may lose stability on small samples [35].

Wrapper methods evaluate the data distribution that results from applying the model that generates the target variable to the original data. Wrapper methods implement genetic algorithms [36,37,38], the particle swarm optimization algorithm [39], the Boruta method [40], the top-down greedy search algorithm [41], and other searching algorithms [42]. Recent publications have shown that wrapper methods can successfully be applied on small samples, especially in eliciting significant groups of genes [43]. However, their fundamental feature is their heuristic nature, which implies that the methods underlying them tend to have no strict mathematical basis. Thus, the results of ranking the features obtained by these methods are compared empirically rather than formally, which may be undesirable in terms of generalizability within the framework of evidence-based medicine.

It is the SHAP (SHapley Additive exPlanation) method that stands out in this respect. The SHAP feature selection method, proposed in [44], is based on Shapley values and was first used in game theory to determine how much each player in a collaborative super-additive game has contributed to its success. The SHAP method has been increasingly applied in feature ranking problems [45,46,47]. However, SHAP values do not only depend on the model, but also on the input data distribution, and even the features that are not used by the model in any way can have non-zero SHAP values. In particular, SHAP values are sensitive to high correlations among different features [48]. The applicability of SHAP to small samples does not appear to have been amply studied. The definition of SHAP is not clear about how exactly the average value *E*[*f*(*x*)|*S*] of the prediction algorithm *f*(*x*) is calculated when fixing the subset of features S under the conditions of a data sample limited in size. Additionally, Ref. [49] also showed that SHAP values and variable rankings based on them fluctuate when using different background datasets acquired from random sampling, and this fluctuation increases with the background dataset size decreasing.

Most of the methods discussed above are frequentist methods, in that they consider all parameters to be fixed and the data to be random. Conversely, Bayesian methods consider that both all parameters and the data are random (and therefore will have distributions). The very definition of Bayesian statistical methods reveals their significant advantage: they are not based on the assumption of big sample sizes or on the theorems about the limiting behavior of distributions. Therefore, they can be used with any sample size and take into account some additional information about the problem, which makes their conclusions more reliable (in the case of useful inductive bias), or estimates the degree of uncertainty of conclusions in the case when there is no additional information.

For example, Ref. [50] builds a generic approach for ensemble FR based on Bayesian models, which in many ways resembles the Boruta wrapper method [40]. In [51], the authors compare three Bayesian ranking methods for categorical predictors using a specialized Gibbs sampler. The authors in [52] built the minimum submodel from all possible predictors, with the performance matching the reference model within a standard error. To avoid overfitting, the submodels are compared to the reference model in cross-validated prediction accuracy, via the efficient Bayesian approximation of leave-one-out cross-validation. Ref. [53] developed a modification of the Bayesian variable selection method, the performance of which was compared to the Lasso method [31] through an extensive simulation study.

The fundamental results of mathematical statistics [54] suggest two ways to organize Bayesian methods for ranking features. First, almost any algorithm for ranking variables can basically be applied to Bayesian models. Second, any classical parametric model can be made Bayesian by introducing some distribution of the parameters *p*(*w*), as well as formalizing the model itself in the form of a likelihood function *p*(*y*|*x*,*w*). The latter is obtained by analyzing the formula that specifies the prediction and error functions. Recent papers related to the extraction of features on smaller samples using Bayesian methods have shown the vast majority of researchers to follow the first option, that is, to apply the algorithm for ranking variables to a ready-made Bayesian model.

In our work, we propose a wrapper method in which we convert the model to a Bayesian model as the first stage of the feature ranking pipeline.

The task of the method is to rank the features of a specific dataset processed by a specific model. We train the selected model on a specific dataset. The result is a trained model $M_{\hat{\theta }}$. This is followed by the Bayesianization procedure, which implies the following: each specific prediction of the model $M_{\hat{\theta }}$ is calculated while adding Gaussian noise to the model parameters (should the model parameters allow this addition) or with another randomization method. For example, when using a tree-type model, a non-terminated transition to descendant nodes is performed in randomly selected ancestor nodes. The result is a Bayesian model $M_{\theta_{\varepsilon }}$, $\theta_{\varepsilon }$, which provides sampling from probability distributions $p\left(y|x\right)$ and $p\left(y|x_{-i}\right)$ instead of deterministic outputs $M_{\hat{\theta }}(x)$. The resulting samples are used to calculate feature ranking based on the Kullbach–Leibler divergence estimate.

Our contribution is as follows:

(1) Our method, unlike the alternatives described above, such as the Vendi Score [18] and MRMR [24], enables feature ranking based on a direct measure via the Kullbach–Leibler divergence $KL\left(p\left(y|x\right)|p\left(y|x_{-i}\right)\right)$ of the effect of feature absence on model prediction.
(2) Since our method uses a sampling of predictions based on randomized model parameters, it is significantly less dependent on the amount of data.
(3) Due to model randomization, our method is applicable to both Bayesian and non-Bayesian models.
(4) In our method, unlike RFE [41], the model is trained only once.

The rest of the paper is organized as follows. In Section 2, we present the theoretical substantiation of the developed method, the accepted metrics for evaluating its effectiveness, and describe the datasets used in the experiments. In Section 3, we present and discuss experimental estimates of the statistical stability of the developed method on small samples, as well as the results of its comparison with other methods. Section 4 concludes the work and presents the prospects for further research.

## 2. Materials and Methods

### 2.1. Theoretical Substantiation of the Developed Method

First, we introduce some notations and conventions. As we consider supervised learning problems, we let *D* stand for a dataset containing sets of pairs of the form (feature vector, target value). For a given example from the dataset *D*, *x_i_* denotes the *i*-th value of the corresponding feature vector or the *i*-th feature, depending on the context, $x_{-i}$ denotes the feature vector without an *i*-th value, and *y* stands for the target value, the class label, or a real number. Our method is a wrapper method for supervised learning models; thus, we have a training sample $D=\;\{\left(x_{i},\;y_{i}\right){\}}_{i=1}^{N_{d}}$. Our method uses Bayesian models; hence, there is a distinction between the distribution p(y|x), which stands for the output distribution of the algorithm with the input features x, and the distribution $p\left(y|x_{-i}\right)$, which refers to the same, but without considering the feature with index i. In the perspective of this analysis, $p\left(y|x\right)$ and $p\left(y|x_{-i}\right)$ will always refer to distributions of Bayesian model predictions, the full forms of which are $p\left(y|x,\;M\right),\;p(y|x_{-i},\;M).$ However, including *M* for the model would make the formulas hardly readable. Also, for some derivation, we might change it to p(y|x,θ) to explicitly highlight the model parameters *θ*. The prior distribution of parameters will be denoted as $p\left(\theta \right)$, and the posterior one as p(θ|D).

To specify the feature relevance, we consider the axiomatics from [55]: the feature $x_{i}$ is considered relevant if there is such a value $x_{i}=c$ of that feature that for any value of the target variable y=b, the following is true:

(1) $$p\left(x_{i}=c,\;S\right)\neq p\left(S\right)$$

where *S* is some assignment of values to some subset of variables that does not contain the attribute $x_{i}$.

The above condition is intuitively clear as it points out that the feature is relevant if it somehow affects the prediction, but it appears to have limitations: it does not give any quantitative description of feature relevance, and the conditions imposed on the features are not strong enough to implement reasonable quantitative estimates. To overcome these limitations, we propose a method for ranking variables in Bayesian models, which is uniformly suitable for arbitrary Bayesian models while fully corresponding to the above condition. We will also show that the model having to be Bayesian is not a limitation, as we can convert classically trained models into Bayesian. The method consists of identifying the differences between the predictive distribution $p\left(y|x\right)$ and the incomplete predictive distribution $p\left(y|x_{-i}\right).$

We use the Kullback–Leibler divergence KL(p|q) as a difference measure [56]:

(2) KL(p|q) =∑ p(x) log{p(x)/q(x)}

where *p* and *q* are probability distributions.

In the case of a discrete distribution at the output of the model, $KL\left(p\left(y|x\right)|p\left(y|x_{-i}\right)\right)$ refers to the amount of information lost when replacing the distribution $p\left(y|x\right)$ with $p\left(y|x_{-i}\right),$ which is a fairly intuitive criterion for the information content of a feature. This interpretation can be directly applied to the case of a continuous output distribution because the difference in the entropy is still defined correctly (unlike the entropy itself).

To estimate $KL\left(p(y|x)|p\left(y|x_{-i}\right)\right)$, we first obtain $p\left(y|x\right)$ and $p\left(y|x_{-i}\right)$. In practice, even when we have a complete description of the model, the exact analytical formula for $p\left(y|x\right)$ can often be intractable or computationally hard, and such cases would require Monte Carlo sampling. For $p\left(y|x_{-i}\right)$, we can also use Monte Carlo estimation by replacing $x_{-i}$ in the feature vector *x* with its alternative values from the dataset. The effects of a smaller dataset are compensated by the fact that we do not sample based only on its values, but also on the model parameter distribution.

**Figure 1:**
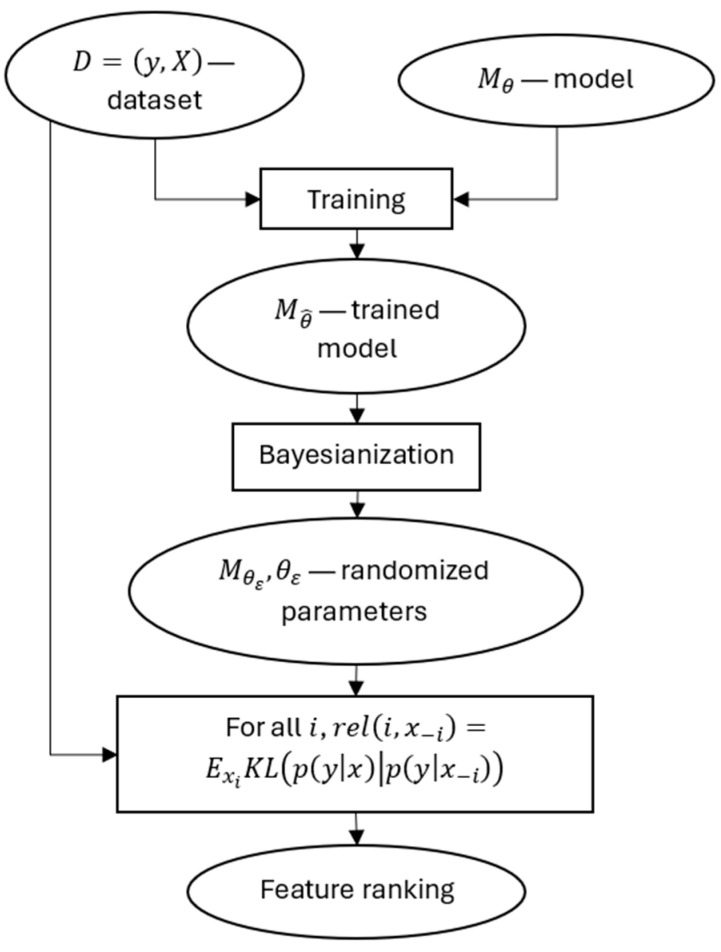
The scheme of the proposed method.

The scheme of the method is shown in Figure 1.

For such models as logistic regression and ensembles of decision trees, we can use shortcuts to avoid relying solely on Monte Carlo methods, which reduces computational costs and further improves the stability on smaller samples. There shortcuts are discussed below.

### 2.2. Analytical Shortcuts for Logistic Regression

Exact Bayesian derivation of the posterior distribution to the parameters is impossible for logistic regression, as the resulting integral is inexpressible. Hence, we use the method of variational approximation. Namely, we can approximate p(w|D) by the multidimensional Gaussian distribution q(w).

Thus, we perform an analytical derivation for $KL\left(p\left(y|x_{-i}\right)\right)$ for Bayesian logistic regression. First, we need an expression for the incomplete predictive distribution $p\left(y|x_{-i}\right)$. It is obtained based on the elementary properties of the conditional probabilities:

(3) $$p\left(y,x_{i}|x_{-i}\right)=p\left(y|x_{-i},\;x_{i}\right)p\left(x_{i}\right)=p\left(y|x\right)p(x_{i})$$

Marginalization of $p\left(y,x_{i}|x_{-i}\right)$ by $x_{i}$ provides us with the following:

(4) $$p\;=\;\int p\left(y|x\right)p\left(x_{i}\right)dx_{i}$$

In logistic regression we have

(5) $$p\left(y|x,\;\theta \right)=\sigma \left(x\cdot \theta \right)$$

where σ is the sigmoid function, and θ is the weights vector of logistic regression.

To estimate the integral (4), we use the following probit approximation:

(6) $$\sigma \left(x\cdot \theta \right)\approx \Phi \left(x\cdot \theta \right)$$

Then, we get the following derivation:

(7) $$p(y=1|x)\;=\int p\left(x,\theta \right)q\left(\theta \right)d\theta \;=\int \Phi \left(\theta \cdot x\right)N\left(\theta_{MAP},\;\Lambda \right)d\theta =\;\int \Phi (a)N(a|\theta_{MAP}\cdot x,\;x^{T}\;\Lambda \;x)da\;=\Phi (\left(\theta_{MAP}\cdot \;x\right)/\sqrt{1\;+\;x^{T}\Lambda \;x}),\;$$

where $\theta_{MAP}$ is the value of the weights vector obtained after variational Bayesian inference, called the maximum a posteriori estimate, and *Λ* is a corresponding covariance matrix.

For marginalization, we also perform analytic inference:

(8) $$\begin{array}{cc} \hat{p}\;=\;p(y\;=\;1\;|x_{-i})\; & =\int p\left(y=1|x\right)p\left(x_{i}\right)dx_{i}\\ & =\int \Phi \left(a\right)N(a\;|\theta_{MAP}\cdot \;x,\;x^{T}\;\Lambda \;x)p(x_{i})da\\ & =\int \Phi (a)N(a|\theta_{MAP}\cdot \;\hat{\theta },\;{\hat{\theta }}^{T}\;\Lambda \;\hat{\theta })da\;=\Phi (\left(\theta_{MAP}\cdot \;x\right)/1\;+\;\;\Lambda \;x \end{array}$$

where $\hat{x}$ is the feature vector x, in which $x_{i}$ is replaced by $Ex_{i}$.

In cases where marginalization (4) is not tractable, but the derivation like (7) is, $p\left(y|x_{-i}\right)$ can be estimated by the Monte Carlo method:

(9) $$p\left(y|x_{-i}\right)\approx \frac{1}{\left|D\right|}\;\Sigma_{x\in D}\;p(y|x_{-i},\;x_{i})$$

where $\left|D\right|$ is the size of the dataset D.

Substituting (7) and (8) into (2), we get the following expression for the KL divergence:

(10) $$KL\left(p\left(y|x\right)|p\left(y|x_{-i}\right)\right)\;=\;p\cdot log\;\left(p/\hat{p}\right)\;+\;\left(1-p\right)\cdot log\;\left(\left(1-p\right)/\left(1-\hat{p}\right)\right)$$

where $p=p\left(y|x\right),\hat{p}=p\left(y|x_{-i}\right)$ are computed by Formulas (7) and (8).

The resulting estimate is of independent significance, as it can calculate the relevance of a feature for the prediction at a specific value of *x*, which opens up opportunities for using our method for local explainability. To derive a criterion for the overall relevance of a feature, we average the following:

(11) $$rel\left(i\right)=\;E_{x}rel\left(i,\;x\right)$$

where $rel\left(i,x\right)$ refers to Formula (10).

The resulting criterion avoids sampling the predictions of logistic regression and proceeds directly to averaging over the dataset D.

### 2.3. Shortcuts for Decision Tree Ensembles

Decision trees can be made Bayesian in a large variety of ways, but we choose here the minimalistic way inspired by the missing value imputation method in the C4.5 algorithm [23].

First, we have the following formula:

(12) $$p\left(y|x,T\right)=p_{leaf_{T}\left(x\right)}$$

where $p_{leaf_{T}\left(x\right)}$ is the probability assigned as a result of training to the leaf where x comes by its decision path.

To obtain $p\left(x_{-i},T\right)$, we use the procedure from the C4.5 algorithm. Instead of following the full decision path for an example x, both directions are chosen in the nodes where the feature $x_{i}$ is used, determining each of the directions with the probability proportional to the number of samples in in the node:

(13) $$p_{left}=\frac{n_{left}}{n_{left}+n_{right}},\;p_{right}=1-p_{left}$$

**Figure 2:**
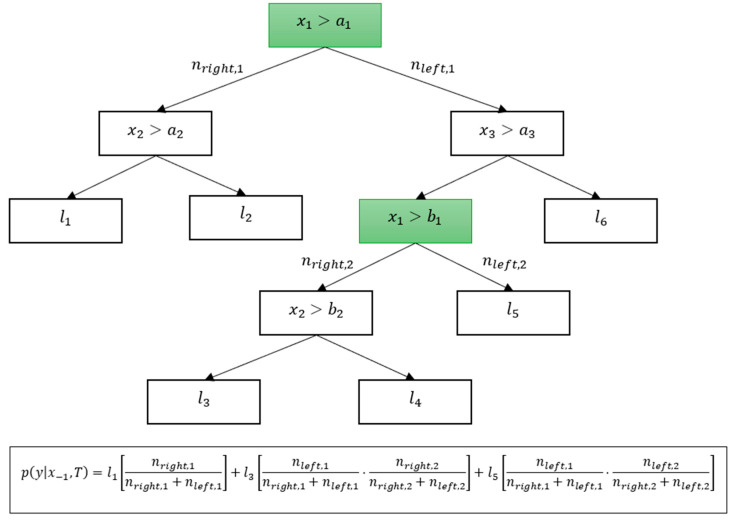
An example of the computation of a Bayesianized tree.

By considering all such paths, we obtain the set of all the leaves of the tree, consistent with the values of the features of x except $x_{i}$ and weighted proportionally to typicality, and we obtain the following estimate for $p\left(y|x_{-i},T\right)$:

(14) $$p\left(y|x_{-i},\;T\right)=\sum_{l\in leaves\left(x_{-i}\right)}p_{l}\cdot p\left(l\right),$$

where $p_{l}$ is the probability assigned to leaf l, and $p\left(l\right)$ is the probability of the path leading to l, which is computed as the product of the expressions in (13) for each node with $x_{i}$ in it, whereas $leaves\left(x_{-i}\right)$ is the set of all leaves, consistent with x except for $x_{i}$. An example of such a computation is shown in Figure 2 below.

Following the above manipulations, Expression (10) can be used directly.

### 2.4. Proposed Method as Related to SHAP on Larger Datasets

For the ablation to the conditions of classical statistics, we estimate the asymptotic behavior of the obtained criterion in the limit of large samples. Using the Bernstein–von Mises theorem [57], we estimate the posterior distribution (and, hence, its variational approximation), as follows:

(15) $$p\left(D\right)\approx \delta (w\;-\;w_{MAP})$$

where $w_{MAP}={argmax}_{w}p(w|D)$. Notably, on the other hand,

(16) $$p\left(y|x_{-i}\right)\approx \int \Phi \left(a\right)\delta \left(a-w_{MAP}\cdot \;\hat{x}\right)da\approx \Phi (w_{MAP}\cdot \hat{x})$$

Thus, we get a result close to the classical one for logistic regression, while marginalization becomes equivalent to filling in the missing feature with its mean value.

For further evaluation, we note that $H\left(p\left(y|x_{-i}\right)\right)$ does not depend on $x_{i}$, so (11) can be written in a more convenient form for estimating asymptotics:

(17) $$rel(i)\;=\;E_{x}[H\left(p\left(y|x_{-i}\right)\right)\;-\;H\left(p\left(y|x\right)\right)]$$

We evaluate the relevance of a particular feature $x_{i}$ using three methods, namely classical logistic regression, SHAP applied to it, and Bayesian logistic regression. The relevance for Bayesian logistic regression is calculated by Expression (17). The native expression for relevance based on classical logistic regression is

(18) $${rel}_{cl}(i)=|w_{i}|,$$

and, for SHAP applied to classical logistic regression, it looks as follows:

(19) $$rel_{SHAP}\left(i\right)=\Sigma_{\left\{S\subset \;\left\{1,\ldots ,N\right\}\backslash \left\{i\right\}\right\}}\frac{\left|S\right|!\left(\left|N\right|-\left|S\right|-1\right)!}{|N|!}\;\Delta_{f}(i,S)$$

where

(20) $$\Delta_{f}(i,\;S)=E[\Phi \left(w\cdot x\right)|x_{S\cup \left\{i\right\}}]-E[\Phi (w\cdot x)\;|\;x_{S}]$$

Notably, $\left|{\left(w_{MAP}\right)}_{i}\right|\to \infty \Rightarrow \;H\left(p\left(x\right)\right)\to \;0$ almost everywhere, and $\left|{\left(w_{MAP}\right)}_{i}\right|$ is a modulus of i-th coordinate of $w_{MAP}$ (21).

From (21), it follows that

(21) $$rel(i)=E_{x}\;[H(p(y|x_{-i}))-H(p(y|x))]=E_{x}\;[H(p(y|x_{-i}))-E_{x}\;[H(p(y|x))]$$

increases because H(x) is non-negative everywhere, so $E_{x_{-i}}rel(i)$ is also. Thus, in the limit, (18) positively correlates with (17).

It is noteworthy that (17) positively correlates with (20) for similar reasons.

The analysis performed confirms that, in the limit of large samples, the ranking results obtained using the proposed algorithm correlate with those of the classical methods of classical logistic regression and SHAP.

### 2.5. Bayesianization Procedure Analysis

Essentially, the method of Bayesianization is to randomize the parameters of the model to make its predictions into a distribution. This is done both for models with additive parameters (generalized linear models, neural networks, etc.) and with non-additive ones (Random Forest), but with some changes. The main goal at this stage is to obtain, instead of $p\left(y|x\right)=\delta \left(y-f\left(x\right)\right)$, a distribution that will not take us out of the “training” zone of the model, but for which the KL divergence will be calculated well.

In the additive case with the model $y=f\left(x,\theta \right)$, we add to the parameters the Gaussian noise $\varepsilon ∼N\left(0,\sigma I\right)$, where σ is the selected parameter (noise variance), and I is the n×n identity matrix, where n is dimension θ, resulting in $y|x∼f\left(x,\theta +\varepsilon \right)$. The validity of the method follows from Taylor expansions for the moments of functions of random variables [58]:

(1) θ+ε∼N(θ,σ).
(2) $f\left(x,\;\theta +\varepsilon \right)\approx f\;+\;\left(x,\;\theta \right)\cdot \varepsilon +\frac{1}{2}\frac{\partial^{2}f}{\partial \theta^{2}}\left(x,\;\theta \right)\cdot \varepsilon^{2}$, the local behavior of the function based on its Taylor expansion.
(3) $E\left[f\left(x,\theta +\varepsilon \right)\right]\approx f\;+\;\frac{\partial^{2}f}{\partial \theta^{2}}\left(x,\theta \right)\cdot \sigma_{\varepsilon }^{2}$—the bias introduced into the output is quadratic in $\sigma_{\varepsilon }$, which guarantees its smallness for small $\sigma_{\varepsilon }$.
(4) $var\left[f\left(x,\;\theta +\varepsilon \right)\right]\approx {\left(\frac{\partial f}{\partial \theta }\left(x,\;\theta \right)\right)}^{2}\cdot \sigma_{\varepsilon }^{2}+\frac{1}{2}{\left(\frac{\partial^{2}f}{\partial \theta^{2}}\left(x,\;\theta \right)\right)}^{2}\cdot \sigma_{\varepsilon }^{4}+\left(\frac{\partial f}{\partial \theta }\left(x,\;\theta \right)\cdot \frac{\partial^{3}f}{\partial \theta^{3}}\left(x,\;\theta \right)\right)\sigma_{\varepsilon }^{4}$—the quadratic and biquadratic dependencies on $\sigma_{\varepsilon }$ guarantee that the scatter of the predictions around $E\left[f\left(x,\theta +\varepsilon \right)\right]$ will be small for small $\sigma_{\varepsilon }$.

We choose values of $\sigma_{\varepsilon }$ around $10^{-2}$, so both the bias and variance are small, but not negligible.

In the non-additive case, randomization becomes more diverse, but we use for tree-like models the method from the C4.5 algorithm, which just averages predictions by all leaves consistent with x except for $x_{i}$. The decision tree is a locally constant function, so averaging over subsets of leaves does not make its predictions out of the domain.

### 2.6. Datasets and Metrics

In our experiment, we used two synthetic datasets, two publicly available datasets describing the symptoms of COVID-19 disease, and three standard datasets for benchmarking feature ranking.

Both synthetic datasets were generated based on the function “make_classification” from the Python 3.12 library sklearn, which is the standard choice for checking the quality of feature ranking [59]. The first dataset, which we refer to as “sklearn_small”, contains 50 features, 15 of which are informative, 8 which are redundant, and 3 which are the duplicates of others. The other features are essentially Gaussian noise. The second dataset, that we denoted “sklearn_large”, consists of 300 features, only 25 of which are informative, 10 which are redundant, and 5 which are the duplicates of others.

The first public dataset [60] is based on publicly released data from the Israeli Ministry of Health, and is composed of the records from 51,831 tested individuals (of whom 4769 were confirmed to have COVID-19), from 22 March 2020 through 31 March 2020. Based on these data, the authors [60] developed a model that predicts COVID-19 test results using eight binary features (sex, age below or above 60 y.o., any known contact with an infected individual, and five initial clinical symptoms). In our experiments, we used all 16 features from the dataset instead of eight.

The second public dataset [61], provided by the Mexican government, contains anonymized patient-related information, including pre-conditions. The raw dataset consists of 21 unique features and 1,048,576 unique patients. The target variable was the information about the death of a patient, based on the column “date of death”.

The first standard dataset [62] is a heart disease dataset which contains 11 features about patients. The second standard dataset [63] is a heart failure prediction dataset with 13 clinical features. The third dataset [64] refers to red wine quality. It contains 11 features about wine and its quality. All datasets contain both categorical and continuous features. Table 1 summarizes the properties of all datasets.

A smaller dataset size was emulated by randomly sampling the original datasets to the desired number of samples. If the size required for the experiment exceeded the size of the dataset (which was only the case in Heart1), then bootstrapping was used.

As there appears to be no gold standard in ranking problems, and in accordance with the condition (1), an essential indicator for assessing the quality of the proposed method is its statistical stability compared to other ranking methods. For this assessment, we used the following metrics:

Self-consistency was defined as follows:

(22) $$SC(n)\;=\;E_{\left(X_{m<n},\;X_{n}\right)}\;Corr(R[X_{m<n}],\;R[X_{n}])$$

where $R[X_{m<n}]$ and $R[X_{n}]$ are the rankings achieved from subsamples $X_{m<n}$ and $X_{n}$ of the sizes m and n, respectively. This metric evaluates the mean correlation between the rankings achieved by the independent subsamples of the size n and the sizes of m<n. This implies that, when working with different samples from the general population that have the size as given or smaller, the resulting rankings should not differ radically. The closer the value of this metric to 1, the more similar the obtained rankings are.

Monotonicity, defined as

(23) $$M\left(n\right)=E_{\left\{\left(X_{m<n}\subset X_{n}\right)\right\}}\;\;Corr(R[X_{m<n}],\;R[X_{n}])$$

implies that when working with the samples from the general population with one being the extension of the other, and thus having different sizes, the resulting rankings in general should not be crucially different. On the other hand, the differences should decrease with the increasing volumes of pairs. Basically, this metric measures the extent to which the feature ranking stays the same when we expand the data.

Consistency with classical methods, defined as

(24) $$MutualConsistency(1,2,n)=E_{X_{n}}\;Corr(R_{1}[X_{n}],\;R_{2}[X_{n}])$$

means that under the most suitable conditions for classical methods (that is, with sufficiently large samples), the results obtained using the proposed method should be consistent with those of classical methods.

Verification of the presence of the above properties requires a criterion Corr for comparing the lists of ranks. For this criterion, we chose the Kendall correlation coefficient as one of the most frequent and natural ways to evaluate the ranking similarity; thus, we can evaluate the rank consistency.

To quantify the applicability of our method, we designed an experiment for evaluating quality improvements and maintenance, following the benchmarking methodology [65]. It is performed on all dataset–model pairs as follows:

(1) A total of 20% of the data goes into the validation sample. From the remaining part, a fixed number of samples (10, 100, or 1000) is selected randomly without replacement into the training dataset.
(2) The model is trained on the remaining part of the data, and the f1-score is calculated on the test sample.
(3) The list of the top n relevant features is obtained with the trained model and the training dataset (in the case of filter methods—only with the dataset).
(4) The model with the same hyperparameters is trained in the same way as in step (2), but only with the features selected in step (3) left. Then, the f1-score on the test sample is calculated.
(5) The ratio of the f1-scores from step (4) and step (2) is calculated.

This experiment was conducted for each model and each dataset, for 10, 100, and 1000 examples in the training data, and with 1, 3, 5, 7, 9, and 12 features left. To ensure statistical stability, we performed the experiment 30 times in each configuration and averaged the results.

## 3. Experimental Results and Discussion

### 3.1. Quality Improvement and Maintenance Experiments

The total results of the experiment for quality improvement evaluation are summarized into a table of the size of 75 × 35 cells. Therefore, we present only the most illustrative parts in Table 2, Table 3 and Table 4. The complete results are included in Appendix A, Table A1, Table A2, Table A3 and Table A4.

For brevity, the tables below show only the top three methods by model quality on the test sample. Some abbreviations were used: “embed” instead of “embedded_ranking”, “our” instead of “our_method”, and the rest of the names did not display the “_ranking” marker.

As Table 2 shows, in most cases, our method and SHAP provide the highest ratios of the metric after filtering to the original in the case of tree models, which means that both methods tend to be the most efficient to select the most relevant features for a particular model. However, the tables show that, while in the case of XGBoost, SHAP is comparable to our method, on Random Forest, our method appears to be more efficient in many more cases. This might mean that our proposed Bayesianization method is preferable on ensemble models where predictors are independent rather than on boosting, where they are dependent.

As Table 3 shows, when our method is used on linear models, it manifests the best results in most cases, showing its power in selecting the features specifically relevant for the model.

As Table 4 shows, the top performance was lost on the dataset “sklearn_small” with a high number of redundant features, and embedded methods together with MRMR ranking prevailed. This may indicate a direction for improving our model, because it shows that redundancy in features tends to affect the performance. The effectiveness of embedded methods is explained by the high robustness of tree-like ensembles to noisy redundant features, which reduces their influence. The effectiveness of MRMR on large datasets is explained by the fact that the correlation between noise features and the target will be near-zero, and those features will not be selected. On smaller samples, its effectiveness is more surprising but can be partially explained by the fast convergence of correlation estimation with Gaussian noise. On the other hand, our method focuses on the influence of features on the model predictions, and the model might capture spurious relations. This is partially mitigated by the Bayesianization scheme, which reduces the influence of irrelevant features, but it seems that in that case, the current scheme is too simple for a purely random features case.

### 3.2. Consistency Experiments

As SHAP appears to be the closest to the proposed method, we conducted consistency experiments with it. For ablation, we used classical logistic regression and Bayesian logistic regression embedded methods of feature ranking.

**Figure 3:**
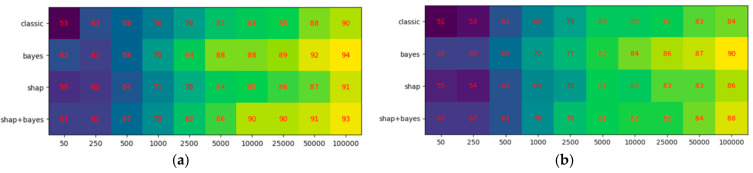
Results of models on the datasets: (**a**) first synthetic, and (**b**) second synthetic.

Using Expressions (17)–(19), we calculated the mean percent of features in the top ranking that are truly relevant for our synthetic dataset. The results are shown in Figure 3a,b, where our method is denoted as “bayes”. The mean percent was evaluated by generating 100 examples of synthetic datasets of different sizes (horizontal axis), ranking them, and then averaging the mean percent of features at the top of the ranking. The variability of results could fluctuate, but never surpassed 2% deviation.

All methods appear to provide effective results; however, our method performed slightly better than the others on both datasets, which appears to confirm the fundamental validity of our method.

We computed the mean values, 25th and 75th percentiles for self-consistency (22), monotonicity (23), and mutual consistency (24) for the rankings obtained by the following methods: classical linear regression, Bayesian linear regression, and SHAP-based ranking for classical and Bayesian regressions. The corresponding expressions were evaluated using the Monte Carlo method based on 100 iterations of the procedure for sampling the subsamples of corresponding sizes, training models on them, and calculating the corresponding rankings and the correlations between them.

**Figure 4:**
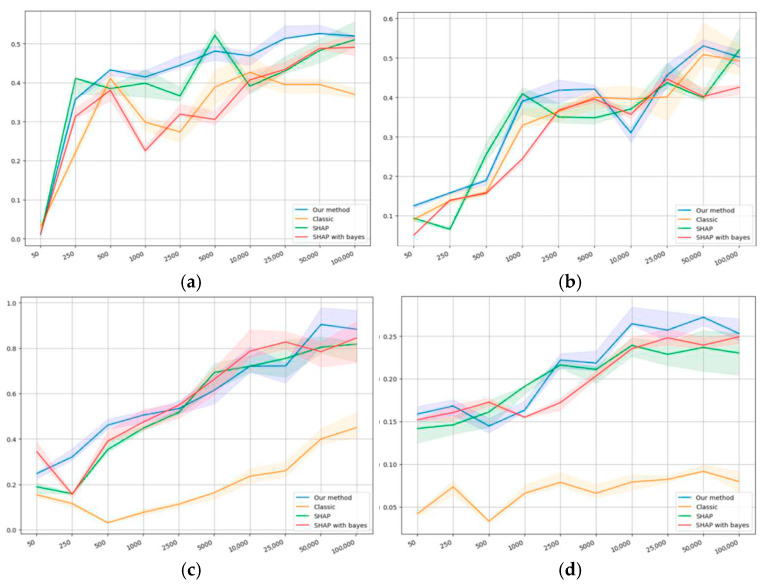
Self-consistency values on the datasets: (**a**) first synthetic, (**b**) second synthetic, (**c**) first public, and (**d**) second public.

The self-consistency results are presented in Figure 4a,d. The horizontal axis shows the dataset size, while the vertical one shows the value of the self-consistency metric (22).

Figure 4 shows that, on the first synthetic dataset at a sample size value of 50, the self-consistency value is extremely small compared to the bigger sample size values. This can be explained by the fact that, for a synthetic dataset of such volume, the sample may be nonrepresentative because of the formal relationship with the target variable. Our model appears to outperform others on average by the values of self-consistency. Figure 4b shows similar results in terms of value; however, it does not demonstrate an anomaly with an extremely small value of 50.

Figure 4c demonstrates a relatively high self-consistency value at a sample size value of 50 compared to the bigger sample size values. This can be explained by the fact that at such volumes, the sample may contain too little information, which results in the model failing to produce sufficiently biased conclusions.

For the classical logistic regression, a dip is manifested in the region of 500−5000, which clearly shows the problem of smaller samples: data volumes have to be large enough to obtain a high level of self-consistency.

SHAP, as a conventional method for quantifying the importance of factors, shows superior results in conjunction with classical regression, and it does not have a dip in the region of 500−1000, which may indicate a higher level of information accumulation than classical logistic regression.

Our method and SHAP, in conjunction with Bayesian regression, appear to show similar results, although our method demonstrates, on average, slightly higher values of the metrics. Both methods are also proven to outperform the others when starting with a sample size of 500.

Importantly, on the second public dataset, all methods show lower self-consistency values compared to the first one; however, our method outperforms the others by at least 0.5−1 units of the correlation coefficient.

The classical logistic regression shows extremely low self-consistency values compared to the other methods, while SHAP with both regressions and our method show similar results.

**Figure 5:**
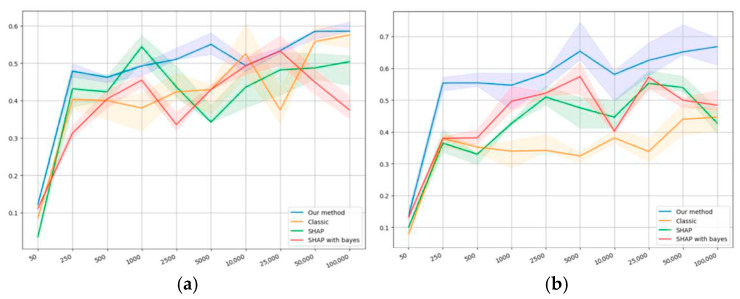

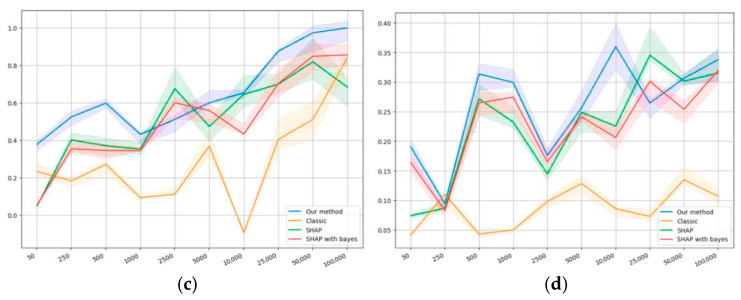
Monotonicity values on the datasets: (**a**) first synthetic, (**b**) second synthetic, (**c**) first public, and (**d**) second public.

Figure 5 compares the top 15 features selected on samples of different sizes from the same dataset. The figure visually demonstrates the self-consistency of our method.

Figure 5a,d presents the results on monotonicity. Figure 5a,b shows that all methods demonstrate similar results on monotonicity, which can be explained by the synthetic nature of the corresponding datasets. Figure 5c manifests that, in terms of monotonicity, the same pattern is observed as with self-consistency: the smallest value is observed for logistic regression, then comes SHAP in relation to classical regression and Bayesian regression, and our method gives the highest value. In terms of monotonicity, the results of applying SHAP to both types of regression are not essentially different.

Our method appeared to demonstrate a significantly greater level of monotonicity, which means that it allows more consistent conclusions about a larger sample based on its subsamples. According to the results on the second public dataset that are presented in Figure 5d, all methods show a significantly lower level of monotonicity compared to the first one. However, the order in terms of the level of monotonicity remains the same, which confirms the previous conclusions. Table 5 presents the comparison of top 15 features selected on samples of different sizes from the same dataset.

**Figure 6:**
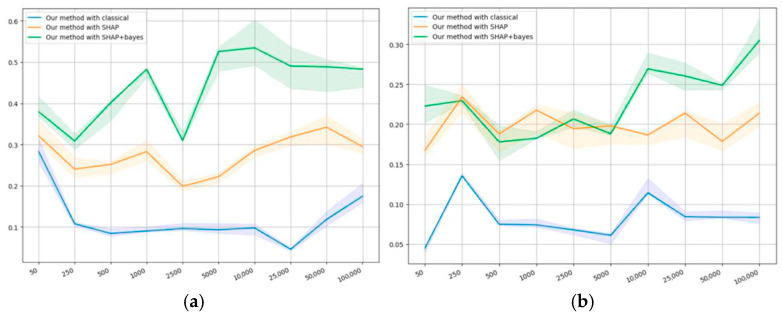
Consistency of the proposed method with the others on the datasets: (**a**) first public, and (**b**) second public.

Figure 6a,b presents the results on mutual consistency.

Figure 6a shows that, on small sample sizes (less than 1000), the mutual consistency of our method is always positive, which means that the methods do not contradict each other. With the growing sample size, the mutual consistency for all methods starts to drop, but then increases again. We suggest the following explanation: on smaller samples, the results of all algorithms can be similar because of the lack of information contained within the sample; however, with the sample size growing, new details may appear and algorithms start to differ.

The agreement with classical regression appears to remain low; however, with the sample growing, the metric’s value eventually tends to increase. The results correlate the closest with SHAP as applied to Bayesian regression, which shows that the methods have some features in common. With SHAP applied to classical regression, the self-consistency is somewhere “in the middle”, which confirms some similarity of our method with SHAP.

Figure 6b shows a similar pattern of correlations: the method correlates least of all with classical regression, and the closest of all with SHAP in relation to Bayesian regression, whereas SHAP with classical regression is “in the middle”. This also confirms the similarity of the results obtained using our method and using SHAP.

We have shown on both synthetic and public datasets that the new ranking method has a slightly higher level of self-consistency on both types of datasets compared to other methods, which is shown in Figure 4a,d. Figure 5a,d demonstrates its significantly higher level of monotonicity on the public datasets and comparable level on synthetic data. Additionally, within the studied sample sizes, our method proves to be the most consistent with the results of applying SHAP to Bayesian regression, less so with applying SHAP to classical regression, and least of all with classical regression. However, in all the three cases, it manifests an increase in consistency with the sample size growing, as shown in Figure 6a,b. On synthetic datasets, the percentage of truly relevant features among the top ranking was computed, and all methods showed similar results; however, our method was slightly better on smaller samples. It is noteworthy that on different datasets, the methods showed different qualities, although an average order was always observed: classical regression, SHAP to classical regression, SHAP to Bayesian regression, and our method.

## 4. Conclusions and Future Work

Based on the Bayesian approach, this paper proposes a solution that allows not only building a methodically justified way of ranking features on small datasets, but also methodically solving the problem of benchmarking the results obtained by various ranking algorithms.

In our work, we propose a wrapper method in which we convert the model to a Bayesian model as the first stage of the feature ranking pipeline. The result is a Bayesian model $M_{\theta_{\varepsilon }}$, $\theta_{\varepsilon }$, which provides sampling from probability distributions $p\left(y|x\right)$ and $p\left(y|x_{-i}\right)$ instead of deterministic outputs $M_{\theta }\left(x\right)$. The resulting samples are used to calculate feature ranking based on the Kullbach–Leibler divergence estimate. In our method, the Kullbach–Leibler divergence is applied in an especial way: instead of quantifying the differences in the distribution of the predictor between the different values of the target, it quantifies the differences in the target distribution on a particular input example, given a particular predictor is removed or not removed. It provides a more model-specific feature ranking procedure, while remaining within the framework of the model-agnostic approach.

We have theoretically justified the validity of our method. We have demonstrated shortcuts for different types of models, improving the computational efficiency of the proposed method. We have theoretically confirmed the equivalence of the classical frequentist approach and the proposed approach.

We have carried out an experimental evaluation of our proposed approach with SOTA methods on a wide experimental base in terms of quality improvement or maintenance after the feature selection procedure, with the top n relevant features left. In most cases, our method manifested the best results, except on the data with a high number of redundant features. We consider this case to be the subject of our further research.

We have carried out an experimental comparison of our proposed approach with the classical method. We have experimentally evaluated the self-consistency, monotonicity, and mutual consistency of the rankings obtained by our methods and the closest SOTA method. All the experiments performed have confirmed our theoretical conclusions: with the growth of the sample, an increasing trend of mutual consistency was observed, and our method demonstrated at least comparable, and often superior, values of self-consistency and monotonicity to other methods.

## Figures and Tables

**Table 1:** Summary of the properties of datasets.

Dataset Name	Number of Features	Number of Examples
sklearn_small	50	30,000
sklearn_large	300	30,000
Covid	16	51,831
Withmeds	21	1,048,576
Heart	11	1026
Heart1	13	919
Winequality-red	11	1600

**Table 2:** The results of the feature ranking methods with tree ensemble models.

			XGBoost			Random Forest		
Dataset	Data Size	Num Features	Top 1	Top 2	Top 3	Top 1	Top 2	Top 3
Heart	10	1	our	embed	shap	our	vsi	mrmr
		6	shap	our	embed	our	vsi	mrmr
		12	shap	permtest	our	embed	shap	our
	1000	1	our	shap	mrmr	our	mrmr	shap
		6	permtest	our	mrmr	permtest	embed	our
		12	embed	permtest	shap	embed	permtest	shap
Heart1	10	1	our	mrmr	shap	our	mrmr	embed
		6	shap	our	embed	shap	embed	our
		12	embed	our	shap	our	vsi	shap
	1000	1	our	mrmr	others	our	permtest	shap
		6	mrmr	shap	embed	vsi	embed	our
		12	mrmr	vsi	shap	shap	mrmr	vsi

**Table 3:** The results of the feature ranking methods with linear models.

			Ridge			ElasticNet		
Dataset	Data Size	Num Features	Top 1	Top 2	Top 3	Top 1	Top 2	Top 3
Heart	10	1	our	vsi	shap	shap	our	vsi
		6	our	shap	embed	our	embed	mrmr
		12	shap	embed	our	vsi	our	permtest
	1000	1	our	mrmr	vsi	our	mrmr	embed
		6	our	permtest	mrmr	our	permtest	mrmr
		12	our	permtest	vsi	our	vsi	permtest
Heart1	10	1	our	embed	shap	our	mrmr	embed
		6	our	vsi	mrmr	embed	our	shap
		12	permtest	vsi	mrmr	our	embed	mrmr
	1000	1	our	mrmr	permtest	our	mrmr	vsi
		6	vsi	our	mrmr	embed	vsi	mrmr
		12	mrmr	our	embed	vsi	our	shap

**Table 4:** The results of the feature ranking methods with ensemble models on the dataset “sklearn_small”.

			XGBoost			Random Forest		
Dataset	Data Size	Num Features	Top 1	Top 2	Top 3	Top 1	Top 2	Top 3
sklearn_small	10	1	permtest	vsi	our	embed	shap	mrmr
		6	mrmr	vsi	permtest	embed	mrmr	permtest
		12	mrmr	embed	shap	embed	vsi	mrmr
	1000	1	permtest	embed	vsi	mrmr	permtest	embed
		6	mrmr	permtest	vsi	mrmr	vsi	embed
		12	our	vsi	shap	vsi	mrmr	our

**Table 5:** Comparison of top 15 features selected on samples of different sizes from the same dataset.

	50 Samples	1000 Samples	10,000 Samples
1	PQ in lead II	P in lead II	Lung surfactant
2	Lung surfactant	Lung surfactant	P in lead II
3	P in lead II	PQ in lead II	PQ in lead II
4	Signs of right-sided heart overload	Signs of right-sided heart overload	Arrhythmia by rate
5	Anticoagulants	Nonspecific intraventricular block	Nonspecific intraventricular block
6	QTc lengthening	QTc lengthening	QTc lengthening
7	Arrhythmia by rate	Arrhythmia by rate	Lopinavir/ritonavir
8	ST segment ischemic depression	Lopinavir/ritonavir	Signs of right-sided heart overload
9	Angle alpha x	Anticoagulants	Anticoagulants
10	Lopinavir/ritonavir	ST segment ischemic depression	Enlargement of the left atrium
11	Enlargement of the left atrium	Enlargement of the left atrium	ST segment ischemic depression
12	Nonspecific intraventricular block	Angle alpha x	Angle alpha x
13	Atrioventricular block (degree)	Atrioventricular block (degree)	Atrioventricular block (degree)
14	Chioroquine/hydroxychloroquine	Bradycardia (1), tachycardia (2), …	Bradycardia (1), tachycardia (2), …
15	Tocilizumab	Tocilizumab	Tocilizumab

## Data Availability

Dataset available on request from the authors.

## Appendix A

**Table A1:** Results of the experiments for tree-based models on the standard datasets.

		Model Name	XGBoost						Random Forest						Decision Tree					
		Method Name	Embedded Ranking	Vsi Ranking	Mrmr Ranking	Permtest Ranking	Shap Ranking	Our Method	Embedded Ranking	Vsi Ranking	Mrmr Ranking	Permtest Ranking	Shap Ranking	Our Method	Embedded Ranking	Vsi Ranking	Mrmr Ranking	Permtest Ranking	Shap Ranking	Our Method
Dataset Name	Data Size	Num Features																		
**Heart**	**10**	**1**	0.998	0.773	0.847	0.722	0.98	**1.17 ***	0.779	0.807	0.793	0.783	0.776	**0.888 ***	1.086	0.857	0.887	0.807	1.086	**1.119 ***
		**3**	1.061	1.061	0.882	0.977	1.035	**1.156 ***	0.838	0.849	0.838	0.911	0.854	**0.992 ***	**1.096 ***	0.929	0.952	0.99	1.07	**1.07**
		**6**	1.089	0.953	1.024	0.99	**1.123 ***	**1.116**	0.875	0.911	0.904	0.88	0.89	**1.023 ***	1.046	0.948	1.002	1.003	**1.053 ***	**1.034**
		**9**	1.074	1.059	1.035	1.071	**1.096 ***	**1.058**	0.933	0.961	0.944	0.948	0.968	**1.033 ***	1.053	1.049	0.961	1.007	**1.056 ***	**1.049**
		**12**	0.993	0.991	0.981	1.032	**1.057 ***	**1.026**	**1.021 ***	1.012	1.001	1.017	1.001	**1.014**	**1.058 ***	1.017	1.006	1.011	1.046	**1.058 ***
	**100**	**1**	0.769	0.815	0.745	0.724	0.838	**0.922 ***	0.727	0.743	0.699	0.677	0.694	**0.892 ***	0.873	0.863	0.813	0.715	0.859	**0.995 ***
		**3**	0.897	0.882	0.874	0.869	0.895	**0.991 ***	0.834	0.828	0.844	0.85	0.816	**0.939 ***	0.967	0.883	0.858	0.86	0.952	**1.015 ***
		**6**	0.943	0.924	0.954	**0.979 ***	0.947	**0.953**	0.943	0.909	0.919	**0.954 ***	0.92	**0.953**	0.979	0.958	0.954	1.008	**1.015 ***	**0.98**
		**9**	0.975	0.969	0.961	**1.0 ***	0.983	**0.968**	0.992	**0.994 ***	0.962	0.979	0.97	**0.967**	0.993	1.012	0.971	**1.031 ***	1.012	**0.959**
		**12**	0.998	0.99	0.984	0.989	**1.004 ***	**0.993**	**1.002 ***	0.991	0.984	1.001	0.993	**0.998**	0.988	1.001	0.984	**1.02 ***	0.996	**0.992**
	**1000**	**1**	0.723	0.701	0.74	0.679	0.783	**0.804 ***	0.717	0.701	0.735	0.665	0.731	**0.774 ***	0.719	0.667	0.686	0.648	0.746	**0.774 ***
		**3**	0.845	0.803	0.844	0.862	0.83	**0.87 ***	0.891	0.79	0.877	**0.899 ***	0.843	**0.856**	0.87	0.838	0.9	**0.921 ***	0.834	**0.854**
		**6**	0.915	0.918	0.937	**0.947 ***	0.936	**0.946**	0.975	0.955	0.955	**0.978 ***	0.946	**0.974**	0.997	0.999	0.98	**1.002 ***	0.989	**0.994**
		**9**	0.956	0.969	0.96	**0.994 ***	0.987	**0.971**	0.992	0.998	**1.001 ***	0.994	0.993	**0.993**	1.006	1.0	1.003	**1.007 ***	1.0	**1.003**
		**12**	**1.002 ***	0.994	0.987	0.995	0.994	**0.985**	**1.001 ***	0.999	0.996	1.0	1.0	**0.995**	1.003	1.003	**1.006 ***	1.004	**1.006 ***	**1.005**
**Heart1**	**10**	**1**	0.989	0.773	1.046	0.789	0.995	**1.186 ***	0.782	0.607	0.812	0.707	0.755	**1.015 ***	0.965	0.827	0.834	0.779	0.965	**1.093 ***
		**3**	1.079	0.899	0.925	0.822	**1.129 ***	**1.092**	0.783	0.791	0.902	0.771	0.743	**0.984 ***	**1.079 ***	0.897	1.012	0.856	1.034	**1.074**
		**6**	1.122	1.016	0.985	0.932	**1.138 ***	**1.122**	0.969	0.943	0.919	0.863	**0.976 ***	**0.944**	1.024	0.985	0.993	0.924	1.021	**1.047 ***
		**9**	1.039	1.065	1.007	0.999	1.062	**1.155 ***	0.986	0.937	0.964	0.923	0.976	**0.988 ***	1.019	1.031	0.959	0.95	1.03	**1.054 ***
		**12**	**1.042 ***	1.019	0.986	0.956	1.022	**1.038**	0.976	0.991	0.975	0.973	0.985	**1.014 ***	1.055	1.01	0.965	0.931	**1.059 ***	**1.021**
	**100**	**1**	0.773	0.768	0.878	0.769	0.79	**1.005 ***	0.705	0.712	0.783	0.728	0.7	**0.975 ***	0.734	0.732	0.894	0.742	0.752	**1.068 ***
		**3**	0.894	0.803	0.966	0.814	0.868	**1.034 ***	0.801	0.8	0.914	0.774	0.806	**0.973 ***	0.916	0.783	0.935	0.767	0.895	**1.06 ***
		**6**	0.952	0.95	0.971	0.88	0.96	**1.0 ***	**0.956 ***	0.898	0.942	0.885	0.953	**0.953**	0.975	0.933	0.976	0.874	0.971	**1.049 ***
		**9**	0.984	0.95	0.984	0.944	0.967	**0.996 ***	0.973	0.949	0.983	0.929	**0.987 ***	**0.973**	0.969	0.986	0.979	0.906	0.96	**1.038 ***
		**12**	**0.992 ***	0.977	0.985	0.991	0.988	**0.979**	0.985	**0.996 ***	0.993	0.994	0.99	**0.987**	0.973	0.979	0.972	0.974	0.978	**1.0 ***
	**1000**	**1**	0.772	0.772	0.784	0.772	0.772	**0.941 ***	0.744	0.75	0.754	0.762	0.754	**0.935 ***	0.828	0.828	0.828	0.828	0.828	**1.047 ***
		**3**	0.849	0.782	0.95	0.866	0.829	**0.959 ***	0.825	0.808	0.93	0.846	0.81	**0.947 ***	0.986	0.782	0.987	0.892	0.934	**1.052 ***
		**6**	0.953	0.947	**0.965 ***	0.883	0.953	**0.952**	0.953	**0.954 ***	0.944	0.893	0.944	**0.949**	1.005	0.96	0.983	0.844	0.959	**1.041 ***
		**9**	0.967	**0.986 ***	0.977	0.898	0.978	**0.974**	0.956	**0.981 ***	0.977	0.924	**0.981 ***	**0.963**	0.995	0.993	0.986	0.893	0.994	**1.049 ***
		**12**	0.983	0.994	**0.995 ***	0.954	0.992	**0.99**	0.977	0.983	0.987	0.966	**0.993 ***	**0.982**	0.976	**1.01 ***	1.007	0.963	0.995	**0.989**
**winequality-red**	**10**	**1**	1520.033	1729.652	0.547	5769.897	1519.869	**5770.483 ***	13377.531	**17197.868 ***	9040.552	7993.623	9548.139	**7337.792**	0.474	825.162	**1651.445 ***	952.612	0.474	**0.574**
		**3**	**5770.666 ***	0.912	0.868	5769.934	5770.443	**1.362**	0.0	0.0	**2443.174 ***	952.418	357.329	**900.1**	0.275	0.477	**1651.739 ***	0.357	0.69	**0.549**
		**6**	1.267	1072.71	1.475	1.137	1.595	**1072.948 ***	1111.285	1322.371	**1663.641 ***	476.29	465.216	**869.665**	0.456	**0.538 ***	0.463	0.521	0.486	**0.421**
		**9**	1072.607	1072.332	0.982	**1073.438 ***	1072.954	**1072.545**	**1608.405 ***	0.076	1321.305	357.182	0.1	**0.0**	0.509	0.746	0.561	**0.769 ***	0.379	**0.42**
		**12**	1.171	0.738	1.079	**1.293 ***	1.279	**0.933**	**487.926 ***	0.135	0.1	357.237	0.136	**0.1**	0.546	0.634	0.835	0.559	0.536	**0.872 ***
	**100**	**1**	0.605	0.428	0.463	0.584	0.428	**0.767 ***	0.482	0.531	0.579	0.613	0.589	**0.922 ***	0.467	0.335	0.403	0.583	0.421	**0.719 ***
		**3**	0.811	0.724	0.675	1.033	0.758	**1.039 ***	0.84	0.766	0.544	0.787	0.774	**1.091 ***	**0.987 ***	0.652	0.579	0.821	0.875	**0.983**
		**6**	0.906	0.923	0.885	0.934	0.809	**1.038 ***	0.93	1.075	0.846	0.806	0.885	**1.224 ***	0.996	0.872	0.838	**1.068 ***	0.992	**1.054**
		**9**	0.955	0.99	0.984	**0.997 ***	0.984	**0.977**	1.08	1.077	0.944	1.058	1.11	**1.194 ***	**1.074 ***	0.959	0.992	1.035	1.039	**0.945**
		**12**	0.994	1.022	1.0	1.021	0.965	**1.024 ***	1.04	**1.081 ***	1.078	1.015	0.961	**1.074**	1.011	1.032	0.932	0.979	0.973	**1.058 ***
	**1000**	**1**	0.229	0.229	0.198	0.298	0.273	**0.426 ***	0.209	0.199	0.208	0.248	0.177	**0.429 ***	0.213	0.187	0.266	**0.363 ***	0.187	**0.292**
		**3**	0.751	0.429	0.54	0.549	0.694	**0.822 ***	0.587	0.416	0.332	0.33	0.564	**0.864 ***	0.752	0.821	0.769	0.731	0.853	**0.947 ***
		**6**	0.92	0.896	0.795	0.8	0.878	**0.937 ***	0.882	0.984	0.768	0.766	0.945	**0.994 ***	1.005	**1.01 ***	0.968	0.958	0.976	**0.986**
		**9**	0.941	**0.965 ***	0.915	0.939	0.93	**0.942**	**1.041 ***	1.019	1.006	0.906	0.986	**1.015**	1.036	**1.063 ***	1.048	1.051	1.009	**1.003**
		**12**	0.994	0.997	0.996	**1.003 ***	0.998	**1.0**	1.025	0.997	0.993	1.028	**1.047 ***	**1.017**	0.995	1.01	**1.029 ***	1.022	0.992	**1.019**

Note: In Table A1, Table A2, Table A3 and Table A4 the asterisk (*) indicates the element that corresponds to the maximum value in the group.

**Table A2:** Results of the experiments for linear models on the standard datasets.

		Model Name	Logistic Regression						Lasso						Ridge						Elastic Net					
		Method Name	Embedded Ranking	Vsi Ranking	Mrmr Ranking	Permtest Ranking	Shap Ranking	Our Method	Embedded Ranking	Vsi Ranking	Mrmr Ranking	Permtest Ranking	Shap Ranking	Our Method	Embedded Ranking	Vsi Ranking	Mrmr Ranking	Permtest Ranking	Shap Ranking	Our Method	Embedded Ranking	Vsi Ranking	Mrmr Ranking	Permtest Ranking	Shap Ranking	Our Method
Dataset Name	Data Size	Num Features																								
**Heart**	**10**	**1**	0.747	0.877	0.773	0.814	0.761	**0.896 ***	0.711	0.812	0.661	**0.841 ***	0.746	**0.811**	0.564	0.733	0.53	0.476	0.564	**0.752 ***	0.53	0.773	0.753	0.693	**0.821 ***	**0.812**
		**3**	0.968	0.926	0.934	0.831	0.915	**1.024 ***	0.915	0.953	0.92	0.903	0.902	**0.998 ***	1.015	1.016	0.936	0.813	1.005	**1.034 ***	0.955	0.877	0.907	0.783	0.871	**0.99 ***
		**6**	0.935	0.876	0.967	0.917	0.912	**0.998 ***	0.868	0.949	0.957	0.889	0.909	**1.037 ***	1.037	0.982	1.007	0.964	1.045	**1.05 ***	0.98	0.953	0.978	0.926	0.962	**1.031 ***
		**9**	**1.012 ***	0.978	0.946	1.007	0.977	**0.984**	0.941	0.945	0.962	0.957	0.946	**0.989 ***	1.063	1.048	1.021	0.997	**1.069 ***	**1.041**	1.009	0.981	0.999	0.942	1.012	**1.013 ***
		**12**	**1.013 ***	0.994	0.994	1.006	**1.013 ***	**1.013 ***	0.98	0.98	0.982	0.993	0.971	**1.009 ***	**1.056 ***	1.006	1.014	1.007	**1.056 ***	**1.029**	1.003	**1.015 ***	1.004	1.007	1.003	**1.014**
	**100**	**1**	0.784	0.82	0.767	0.769	0.775	**0.926 ***	0.696	0.86	0.849	0.782	0.718	**0.932 ***	0.811	0.83	0.841	0.749	0.841	**0.949 ***	0.755	0.802	0.828	0.79	0.795	**0.932 ***
		**3**	0.847	0.866	0.902	0.849	0.853	**0.942 ***	0.873	0.877	0.911	0.88	0.847	**0.941 ***	0.904	0.919	0.891	0.893	0.901	**0.966 ***	0.865	0.872	0.922	0.896	0.9	**0.945 ***
		**6**	0.933	0.908	0.943	0.934	0.917	**0.977 ***	0.931	0.914	0.948	**0.981 ***	0.91	**0.973**	0.951	0.966	0.974	**0.984 ***	0.951	**0.97**	0.946	0.966	0.973	**0.988 ***	0.94	**0.983**
		**9**	0.969	**0.983 ***	0.973	0.981	0.964	**0.971**	0.981	0.962	0.972	**1.006 ***	0.971	**0.963**	0.983	**0.998 ***	**0.998 ***	**0.998 ***	0.993	**0.971**	0.991	0.996	0.974	**1.005 ***	0.993	**0.975**
		**12**	0.993	0.992	0.991	1.006	0.994	**1.008 ***	1.012	0.991	0.982	1.012	1.012	**1.016 ***	0.992	**1.008 ***	0.999	0.998	0.992	**1.005**	0.988	1.002	0.986	**1.01 ***	0.988	**1.005**
	**1000**	**1**	0.728	0.748	0.783	0.754	0.711	**0.882 ***	0.761	0.837	0.854	0.757	0.668	**0.898 ***	0.724	0.794	0.825	0.754	0.726	**0.902 ***	0.749	0.741	0.824	0.747	0.733	**0.896 ***
		**3**	0.872	0.756	0.827	0.861	0.869	**0.928 ***	0.865	0.791	0.844	0.872	0.89	**0.925 ***	0.891	0.795	0.842	0.864	0.894	**0.928 ***	0.869	0.761	0.858	0.88	0.881	**0.922 ***
		**6**	0.891	0.905	0.91	0.953	0.876	**0.959 ***	0.879	0.906	0.93	0.937	0.89	**0.966 ***	0.906	0.9	0.928	0.957	0.92	**0.963 ***	0.894	0.901	0.914	0.96	0.897	**0.968 ***
		**9**	0.944	0.969	0.935	**0.983 ***	0.938	**0.949**	0.922	0.963	0.933	**0.967 ***	0.934	**0.949**	0.955	0.959	0.946	**0.976 ***	0.958	**0.949**	0.928	**0.981 ***	0.943	0.97	0.936	**0.947**
		**12**	0.973	0.994	0.975	**1.004 ***	0.976	**1.002**	0.964	0.989	0.966	0.986	0.964	**0.997 ***	0.986	0.997	0.993	1.0	0.987	**1.005 ***	0.973	1.0	0.976	0.993	0.973	**1.004 ***
**Heart1**	**10**	**1**	0.87	0.866	0.775	0.831	0.808	**1.136 ***	0.68	0.795	0.834	0.812	0.772	**0.979 ***	0.921	0.497	0.678	0.572	0.921	**1.193 ***	0.893	0.851	0.914	0.815	0.879	**0.935 ***
		**3**	0.874	0.927	1.058	0.901	0.915	**1.103 ***	0.797	0.819	0.926	0.844	0.794	**0.958 ***	1.113	0.816	1.036	0.7	1.11	**1.135 ***	0.889	0.875	0.95	0.828	0.877	**1.033 ***
		**6**	1.007	1.015	0.94	0.873	0.942	**1.035 ***	0.937	**0.953 ***	0.942	0.88	0.919	**0.949**	0.996	1.07	1.004	0.979	1.003	**1.08 ***	**1.006 ***	0.971	0.979	0.958	1.002	**1.004**
		**9**	0.977	1.014	0.945	0.963	0.931	**1.028 ***	0.962	0.966	**0.972 ***	0.925	0.95	**0.955**	1.012	1.037	1.027	1.066	1.011	**1.101 ***	0.995	0.989	0.988	0.966	1.005	**1.029 ***
		**12**	0.973	**1.023 ***	0.977	0.934	0.97	**1.002**	0.986	0.975	0.984	0.964	**0.987 ***	**0.978**	1.009	1.031	1.021	**1.038 ***	1.008	**1.012**	1.003	0.976	1.001	0.992	0.984	**1.016 ***
	**100**	**1**	0.849	0.844	0.869	0.843	0.83	**0.993 ***	0.819	0.792	0.915	0.81	0.836	**0.986 ***	0.796	0.805	0.865	0.835	0.847	**1.004 ***	0.899	0.808	0.842	0.812	0.883	**0.977 ***
		**3**	0.903	0.857	0.969	0.852	0.884	**0.993 ***	0.854	0.813	0.965	0.799	0.871	**0.983 ***	0.931	0.772	0.949	0.819	0.914	**1.008 ***	0.952	0.839	0.973	0.817	0.943	**0.992 ***
		**6**	0.962	0.96	0.988	0.936	0.97	**0.989 ***	0.954	0.949	0.967	0.926	0.951	**0.974 ***	0.992	0.918	0.972	0.884	**1.006 ***	**0.984**	0.973	0.95	0.991	0.906	0.976	**0.996 ***
		**9**	0.989	1.003	0.985	0.943	0.99	**1.019 ***	0.977	0.981	0.985	0.931	0.973	**0.991 ***	0.991	0.989	0.989	0.938	0.993	**1.02 ***	0.985	0.977	0.998	0.914	0.987	**1.01 ***
		**12**	0.997	0.99	**1.009 ***	0.984	1.003	**1.003**	0.981	0.994	0.992	0.981	0.983	**0.997 ***	0.994	1.001	0.998	0.986	1.001	**1.015 ***	0.993	0.989	0.992	0.994	0.994	**1.002 ***
	**1000**	**1**	0.766	0.785	0.839	0.785	0.733	**0.949 ***	0.818	0.785	0.825	0.785	0.799	**0.957 ***	0.78	0.795	0.843	0.795	0.676	**0.957 ***	0.747	0.796	0.833	0.796	0.67	**0.95 ***
		**3**	0.854	0.797	0.944	0.864	0.848	**0.952 ***	0.873	0.805	**0.954 ***	0.879	0.871	**0.952**	0.858	0.819	0.935	0.862	0.854	**0.953 ***	0.874	0.809	0.923	0.854	0.85	**0.953 ***
		**6**	0.953	0.954	**0.963 ***	0.906	0.938	**0.957**	0.952	0.955	**0.97 ***	0.918	0.936	**0.959**	0.945	**0.967 ***	0.952	0.94	0.945	**0.962**	**0.97 ***	0.961	0.96	0.928	0.949	**0.956**
		**9**	0.971	**0.982 ***	0.974	0.909	0.958	**0.981**	0.959	0.986	0.983	0.925	0.951	**0.987 ***	0.971	0.97	0.988	0.938	0.965	**0.99 ***	0.969	0.989	0.979	0.927	0.976	**0.992 ***
		**12**	0.991	0.996	0.985	0.959	0.989	**0.999 ***	0.99	0.997	**1.0 ***	0.959	0.984	**1.0 ***	0.987	0.986	**1.0 ***	0.949	0.982	**0.994**	0.982	**0.998 ***	0.989	0.963	0.991	**0.997**
**winequality-red**	**10**	**1**	0.599	0.435	0.78	0.958	0.599	**1.495 ***	0.089	0.315	0.084	0.168	0.089	**0.344 ***	0.0	0.016	0.021	0.212	0.047	**0.287 ***	3714.286	0.0	0.035	1584.399	3714.286	**3714.554 ***
		**3**	0.973	0.792	0.784	1.228	1.081	**1.835 ***	465.489	455.053	425.803	408.755	**465.579 ***	**455.398**	0.3	0.033	**408.198 ***	0.399	0.281	**0.549**	1970.529	416.792	416.964	**2178.225 ***	1970.529	**385.13**
		**6**	1.103	0.8	0.976	1.006	1.094	**1.487 ***	0.593	**889.758 ***	0.583	0.65	0.56	**435.808**	0.471	**454.704 ***	0.441	0.403	0.513	**0.625**	0.647	**2105.8 ***	1887.274	1569.186	0.649	**0.471**
		**9**	1.049	0.756	1.018	0.87	0.96	**1.089 ***	0.846	**0.951 ***	0.735	0.88	0.874	**0.935**	0.571	454.937	0.58	0.554	0.613	**851.681 ***	0.617	1177.146	**1539.193 ***	0.631	0.617	**0.565**
		**12**	**1.0 ***	**1.0 ***	**1.0 ***	**1.0 ***	**1.0 ***	**1.0 ***	**0.9 ***	**0.9 ***	**0.9 ***	**0.9 ***	**0.9 ***	**0.9 ***	0.6	**0.601 ***	0.6	0.6	**0.601 ***	**0.6**	**0.8 ***	**0.8 ***	**0.8 ***	**0.8 ***	**0.8 ***	**0.8 ***
	**100**	**1**	0.06	0.012	0.078	0.166	0.06	**0.542 ***	0.065	0.0	0.043	0.088	0.065	**0.342 ***	0.0	0.029	0.025	0.089	0.0	**0.479 ***	0.023	0.0	0.0	0.031	0.023	**0.245 ***
		**3**	0.308	0.226	0.317	0.675	0.348	**0.923 ***	0.29	0.168	0.128	0.412	0.315	**0.518 ***	0.312	0.167	0.115	0.355	0.32	**0.614 ***	0.16	0.059	0.102	0.505	0.244	**0.629 ***
		**6**	0.651	0.846	0.705	**0.878 ***	0.614	**0.865**	**0.718 ***	0.702	0.621	0.659	0.616	**0.704**	0.81	0.461	0.367	0.683	**0.862 ***	**0.758**	0.636	0.758	0.521	0.608	0.638	**0.881 ***
		**9**	0.885	0.922	0.914	0.953	0.932	**1.04 ***	0.896	**1.024 ***	0.901	0.918	0.862	**0.892**	**1.006 ***	0.921	0.764	1.003	0.989	**0.87**	0.838	**1.035 ***	0.697	0.963	0.898	**0.962**
		**12**	**1.0 ***	**1.0 ***	**1.0 ***	**1.0 ***	**1.0 ***	**1.0 ***	**1.0 ***	**1.0 ***	**1.0 ***	**1.0 ***	**1.0 ***	**1.0 ***	**1.0 ***	**1.0 ***	**1.0 ***	**1.0 ***	**1.0 ***	**1.0 ***	**1.0 ***	**1.0 ***	**1.0 ***	**1.0 ***	**1.0 ***	**1.0 ***
	**1000**	**1**	0.024	0.0	0.009	0.0	0.008	**0.485 ***	0.0	0.0	0.012	0.0	0.0	**0.501 ***	0.0	0.0	0.0	0.0	0.0	**0.513 ***	0.0	0.0	0.0	0.0	0.0	**0.559 ***
		**3**	0.469	0.283	0.089	0.101	0.481	**0.708 ***	0.503	0.251	0.117	0.206	0.478	**0.765 ***	0.445	0.135	0.036	0.037	0.439	**0.77 ***	0.366	0.178	0.092	0.042	0.316	**0.744 ***
		**6**	0.736	**0.944 ***	0.539	0.513	0.762	**0.782**	0.807	**0.993 ***	0.654	0.554	0.861	**0.811**	0.721	**0.922 ***	0.497	0.381	0.708	**0.805**	0.58	**0.969 ***	0.572	0.53	0.488	**0.776**
		**9**	0.931	**0.961 ***	0.921	0.835	0.929	**0.922**	0.958	**0.973 ***	0.896	0.893	0.955	**0.948**	0.913	**0.988 ***	0.834	0.852	0.906	**0.852**	1.009	**1.038 ***	0.754	0.846	1.035	**0.936**
		**12**	**1.0 ***	**1.0 ***	**1.0 ***	**1.0 ***	**1.0 ***	**1.0 ***	**1.0 ***	**1.0 ***	**1.0 ***	**1.0 ***	**1.0 ***	**1.0 ***	**1.0 ***	**1.0 ***	**1.0 ***	**1.0 ***	**1.0 ***	**1.0 ***	1.0	**1.002 ***	**1.002 ***	**1.002 ***	**1.002 ***	**1.0**

**Table A3:** Results of the experiments for tree-based models on sklearn-generated datasets and on the public datasets.

		Model Name	XGBoost						Random Forest						Decision Tree					
		Method Name	Embedded Ranking	Vsi Ranking	Mrmr Ranking	Permtest Ranking	Shap Ranking	Our Method	Embedded Ranking	Vsi Ranking	Mrmr Ranking	Permtest Ranking	Shap Ranking	Our Method	Embedded Ranking	Vsi Ranking	Mrmr Ranking	Permtest Ranking	Shap Ranking	Our Method
Dataset Name	Data Size	Num Features																		
**covid**	**10**	**1**	0.989	1.003	1.001	1.006	**1.055 ***	**1.044**	0.998	0.995	0.996	0.998	**1.048 ***	**1.041**	0.994	1.012	1.006	1.009	**1.044 ***	**1.042**
		**3**	0.988	0.99	0.993	0.98	**1.037 ***	**1.037 ***	0.996	0.995	0.996	0.993	1.045	**1.046 ***	0.975	0.996	0.973	1.0	**1.024 ***	**1.024 ***
		**6**	0.988	0.991	0.984	0.983	**1.037 ***	**1.037 ***	0.998	0.996	0.995	0.996	**1.048 ***	**1.046**	0.971	0.988	1.004	0.995	**1.02 ***	**1.003**
		**9**	0.983	0.988	0.988	0.978	**1.029 ***	**1.028**	0.997	0.996	0.995	0.996	**1.047 ***	**1.045**	0.958	0.99	1.002	0.978	1.017	**1.023 ***
		**12**	0.982	0.991	0.996	0.99	1.022	**1.033 ***	0.998	0.997	0.999	0.997	**1.048 ***	**1.046**	0.955	0.996	**1.004 ***	0.985	1.002	**1.003**
	**100**	**1**	0.993	0.991	0.993	0.99	**1.041 ***	**1.041 ***	0.99	0.99	0.99	0.988	**1.038 ***	**1.037**	0.994	0.994	1.001	0.998	**1.047 ***	**1.044**
		**3**	0.987	0.987	0.992	0.985	**1.034 ***	**1.032**	0.984	0.989	0.989	0.99	1.031	**1.034 ***	0.989	0.993	1.001	0.989	**1.045 ***	**1.032**
		**6**	0.986	0.986	0.992	0.983	**1.032 ***	**1.032 ***	0.983	0.986	0.986	0.986	**1.035 ***	**1.032**	0.981	0.98	0.996	0.99	**1.035 ***	**1.03**
		**9**	0.988	0.985	0.992	0.983	**1.033 ***	**1.03**	0.987	0.988	0.985	0.989	**1.035 ***	**1.032**	0.979	0.986	0.996	0.986	**1.035 ***	**1.029**
		**12**	0.986	0.991	0.991	0.989	**1.032 ***	**1.029**	0.987	0.99	0.986	0.992	**1.036 ***	**1.033**	0.976	0.986	0.991	0.989	**1.033 ***	**1.026**
	**1000**	**1**	0.969	0.967	0.967	0.966	1.014	**1.015 ***	0.976	0.975	0.975	0.975	**1.026 ***	**1.024**	0.972	0.973	0.973	0.972	**1.022 ***	**1.021**
		**3**	0.967	0.965	0.966	0.971	**1.015 ***	**1.015 ***	0.971	0.973	0.975	0.977	**1.024 ***	**1.024 ***	0.972	0.971	0.971	0.977	**1.02 ***	**1.02 ***
		**6**	0.967	0.964	0.968	0.971	**1.014 ***	**1.013**	0.973	0.974	0.979	0.977	**1.025 ***	**1.024**	0.969	0.969	0.972	0.973	1.016	**1.017 ***
		**9**	0.968	0.966	0.968	0.974	**1.015 ***	**1.014**	0.976	0.975	0.982	0.979	**1.027 ***	**1.023**	0.968	0.962	0.969	0.971	**1.016 ***	**1.015**
		**12**	0.968	0.967	0.97	0.975	**1.016 ***	**1.013**	0.977	0.977	0.982	0.984	**1.029 ***	**1.024**	0.966	0.963	0.965	0.972	1.013	**1.014 ***
**withmeds**	**10**	**1**	0.082	0.093	0.214	**0.785 ***	0.086	**0.086**	**4762.011 ***	4000.195	0.146	4467.687	0.037	**2863.881**	0.12	0.332	0.526	**1.13 ***	0.126	**0.126**
		**3**	0.126	0.221	0.518	**0.785 ***	0.132	**0.132**	**3636.922 ***	1818.697	2222.571	3350.885	2210.907	**0.259**	0.351	0.343	0.483	**1.044 ***	0.369	**0.369**
		**6**	0.672	0.466	0.514	**0.785 ***	0.717	**0.724**	3636.938	3334.131	3333.746	3350.863	2211.127	**5250.59 ***	0.917	0.429	0.901	**1.045 ***	0.945	**0.964**
		**9**	**0.727 ***	0.535	0.605	0.718	0.686	**0.679**	2222.879	2667.294	**4615.69 ***	2978.688	1615.923	**3818.736**	0.853	0.416	1.035	**1.045 ***	0.82	**0.817**
		**12**	0.549	0.57	0.627	**0.689 ***	0.573	**0.499**	**5455.078 ***	2857.899	2857.665	3350.82	1500.615	**3000.712**	0.95	0.944	**1.065 ***	1.025	0.913	**0.715**
	**100**	**1**	0.0	0.104	0.089	**0.73 ***	0.0	**0.0**	0.274	0.065	0.075	**0.73 ***	0.129	**0.0**	0.0	0.018	0.135	**0.734 ***	0.0	**0.0**
		**3**	0.118	0.288	0.268	**0.73 ***	0.117	**0.152**	0.362	0.283	0.192	**0.691 ***	0.414	**0.137**	0.232	0.395	0.382	**0.734 ***	0.244	**0.247**
		**6**	0.565	0.401	0.382	0.73	0.637	**0.866 ***	0.396	0.285	0.358	0.721	0.589	**0.864 ***	0.761	0.5	0.506	0.734	0.799	**0.908 ***
		**9**	0.732	0.471	0.472	0.73	0.745	**0.753 ***	0.453	0.508	0.389	0.708	0.68	**0.771 ***	0.885	0.528	0.521	0.734	**0.916 ***	**0.874**
		**12**	0.778	0.479	0.513	0.73	**0.835 ***	**0.751**	0.418	0.586	0.561	0.673	0.707	**0.744 ***	0.821	0.606	0.642	0.73	**0.858 ***	**0.819**
	**1000**	**1**	0.0	0.068	0.068	**0.693 ***	0.0	**0.0**	0.067	0.146	0.079	**0.73 ***	0.19	**0.0**	0.0	0.14	0.044	**0.736 ***	0.0	**0.0**
		**3**	0.072	0.251	0.307	**0.693 ***	0.118	**0.091**	0.34	0.174	0.31	**0.687 ***	0.249	**0.175**	0.264	0.348	0.476	**0.736 ***	0.277	**0.277**
		**6**	0.327	0.551	0.426	0.693	0.248	**0.785 ***	0.298	0.37	0.434	0.687	0.319	**0.849 ***	0.829	0.344	0.541	0.736	0.87	**0.932 ***
		**9**	0.555	0.55	0.507	0.693	0.648	**0.729 ***	0.401	0.486	0.501	0.675	0.339	**0.679 ***	0.757	0.434	0.707	0.736	0.8	**0.81 ***
		**12**	0.767	0.61	0.615	0.693	**0.81 ***	**0.718**	0.357	0.521	0.615	**0.676 ***	0.499	**0.671**	0.732	0.563	0.698	0.736	**0.779 ***	**0.761**
**sklearn_smal -red**	**10**	**1**	0.702	0.78	0.677	**0.809 ***	0.704	**0.736**	**0.797 ***	0.736	0.752	0.73	0.789	**0.709**	0.879	0.872	0.883	**0.893 ***	0.879	**0.842**
		**3**	0.777	0.726	**0.847 ***	0.816	0.702	**0.722**	**0.894 ***	0.804	0.843	0.818	0.851	**0.665**	0.866	**1.014 ***	0.964	0.963	0.884	**0.796**
		**6**	0.794	0.831	**0.913 ***	0.818	0.796	**0.752**	**0.946 ***	0.865	0.93	0.909	0.886	**0.741**	0.934	1.021	1.002	**1.108 ***	0.928	**0.878**
		**9**	0.891	0.895	**0.973 ***	0.82	0.875	**0.882**	**0.94 ***	0.916	0.939	0.93	0.924	**0.882**	0.97	**1.125 ***	0.959	1.021	1.029	**0.972**
		**12**	0.92	0.9	**0.958 ***	0.848	0.914	**0.88**	**0.965 ***	0.961	0.953	0.934	0.943	**0.938**	0.973	**1.085 ***	1.026	1.074	0.939	**0.914**
	**100**	**1**	**0.661 ***	0.626	**0.661 ***	0.658	0.655	**0.606**	0.63	0.636	0.691	**0.701 ***	0.646	**0.613**	0.653	0.651	0.713	**0.75 ***	0.626	**0.631**
		**3**	0.774	0.74	0.797	**0.807 ***	0.766	**0.613**	0.741	0.739	**0.801 ***	0.792	0.764	**0.608**	0.673	0.746	**0.845 ***	0.828	0.653	**0.661**
		**6**	0.845	0.816	**0.881 ***	0.855	0.85	**0.697**	0.862	0.831	**0.881 ***	0.86	0.845	**0.737**	0.788	0.868	**0.911 ***	0.877	0.793	**0.763**
		**9**	**0.909 ***	0.901	0.907	0.896	0.872	**0.878**	0.907	0.896	**0.914 ***	0.894	0.882	**0.909**	0.868	0.907	0.927	**0.947 ***	0.875	**0.88**
		**12**	0.924	0.938	**0.956 ***	0.932	0.922	**0.914**	0.932	**0.943 ***	0.926	**0.943 ***	0.914	**0.93**	0.921	0.944	0.967	**0.969 ***	0.925	**0.929**
	**1000**	**1**	0.639	0.636	0.619	**0.653 ***	0.615	**0.552**	0.593	0.629	**0.723 ***	0.681	0.623	**0.565**	**0.628 ***	0.625	0.62	0.615	0.617	**0.577**
		**3**	0.716	0.741	**0.773 ***	0.766	0.724	**0.562**	0.724	0.753	**0.81 ***	0.751	0.736	**0.556**	0.736	0.691	**0.738 ***	0.724	0.725	**0.596**
		**6**	0.823	0.823	**0.858 ***	0.833	0.806	**0.718**	0.853	0.863	**0.877 ***	0.825	0.848	**0.729**	**0.843 ***	0.783	0.819	0.807	0.839	**0.715**
		**9**	0.888	0.892	0.902	0.884	0.878	**0.915 ***	0.908	**0.931 ***	0.912	0.918	0.917	**0.923**	0.879	0.856	0.85	**0.907 ***	0.889	**0.887**
		**12**	0.936	0.941	0.935	0.935	0.939	**0.954 ***	0.929	**0.955 ***	0.954	0.932	0.941	**0.946**	0.911	0.915	0.916	**0.93 ***	0.914	**0.92**
**sklearn_large**	**10**	**1**	0.87	0.949	0.856	**0.957 ***	0.87	**0.87**	0.833	0.833	0.82	**0.838 ***	0.756	**0.794**	0.842	**0.895 ***	0.757	0.757	0.842	**0.842**
		**3**	0.914	**0.928 ***	0.858	0.916	0.882	**0.916**	0.783	0.827	0.834	**0.875 ***	0.771	**0.779**	0.93	0.899	0.904	0.873	0.92	**0.965 ***
		**6**	0.976	0.955	0.956	0.913	**0.984 ***	**0.98**	0.875	0.86	0.877	0.901	0.845	**0.959 ***	**0.995 ***	0.986	0.981	0.896	0.983	**0.982**
		**9**	0.905	0.994	**0.999 ***	0.953	0.897	**0.935**	**0.927 ***	0.912	0.897	0.915	0.859	**0.924**	1.005	0.956	0.976	1.0	**1.007 ***	**0.933**
		**12**	**1.107 ***	1.023	0.94	0.968	1.076	**1.101**	0.919	0.924	0.876	0.913	0.89	**0.996 ***	1.056	0.972	0.962	1.021	1.078	**1.117 ***
	**100**	**1**	0.67	0.671	0.685	0.668	**0.691 ***	**0.666**	0.662	0.655	0.654	0.674	**0.676 ***	**0.67**	0.678	**0.714 ***	0.67	0.653	0.678	**0.678**
		**3**	0.734	0.709	**0.766 ***	0.721	0.738	**0.721**	0.692	0.687	0.722	0.669	0.717	**0.732 ***	0.751	**0.759 ***	0.747	0.717	0.744	**0.742**
		**6**	0.734	0.74	0.807	0.765	0.783	**0.886 ***	0.778	0.713	0.756	0.728	0.739	**0.883 ***	0.83	0.776	0.819	0.809	0.834	**0.906 ***
		**9**	0.753	0.762	0.815	0.796	0.801	**0.879 ***	0.804	0.767	0.788	0.753	0.803	**0.871 ***	**0.908 ***	0.816	0.827	0.862	0.893	**0.878**
		**12**	0.791	0.81	0.839	0.814	0.807	**0.961 ***	0.819	0.779	0.835	0.811	0.802	**0.939 ***	0.954	0.824	0.851	0.866	0.934	**0.955 ***
	**1000**	**1**	0.591	**0.65 ***	0.609	0.571	0.62	**0.592**	0.617	0.585	0.589	0.618	**0.632 ***	**0.577**	0.644	**0.646 ***	0.622	0.612	0.612	**0.616**
		**3**	0.636	0.664	**0.69 ***	0.61	0.653	**0.646**	0.634	0.594	0.634	**0.656 ***	0.648	**0.648**	**0.685 ***	0.658	0.683	0.645	0.656	**0.681**
		**6**	0.671	0.661	0.708	0.698	0.711	**0.837 ***	0.675	0.671	0.754	0.753	0.677	**0.85 ***	0.706	0.703	0.77	0.751	0.681	**0.849 ***
		**9**	0.697	0.699	0.779	0.716	0.736	**0.836 ***	0.713	0.722	0.778	0.786	0.715	**0.848 ***	0.749	0.762	0.79	0.761	0.726	**0.841 ***
		**12**	0.704	0.712	0.783	0.772	0.77	**0.898 ***	0.726	0.778	0.811	0.809	0.765	**0.904 ***	0.802	0.799	0.828	0.776	0.778	**0.921 ***

**Table A4:** Results of the experiments for linear models on sklearn-generated datasets and on the public datasets.

		Model Name	Logistic Regression						Lasso						Ridge						Elastic Net					
		Method Name	Embedded Ranking	Vsi Ranking	Mrmr Ranking	Permtest Ranking	Shap Ranking	Our Method	Embedded Ranking	Vsi Ranking	Mrmr Ranking	Permtest Ranking	Shap Ranking	Our Method	Embedded Ranking	Vsi Ranking	Mrmr Ranking	Permtest Ranking	Shap Ranking	Our Method	Embedded Ranking	Vsi Ranking	Mrmr Ranking	Permtest Ranking	Shap Ranking	Our Method
Dataset Name	Data Size	Num Features																								
**covid**	**10**	**1**	**1.0 ***	**1.0 ***	**1.0 ***	**1.0 ***	**1.0 ***	**1.0 ***	**1.0 ***	**1.0 ***	**1.0 ***	**1.0 ***	**1.0 ***	**1.0 ***	**1.0 ***	**1.0 ***	**1.0 ***	**1.0 ***	**1.0 ***	**1.0 ***	1.0	**1.002 ***	**1.002 ***	**1.002 ***	**1.002 ***	**1.0**
		**3**	1.012	1.013	1.022	1.014	**1.057 ***	**1.057 ***	1.013	1.011	1.013	1.008	**1.063 ***	**1.044**	1.017	1.011	1.019	1.013	**1.068 ***	**1.064**	1.001	1.001	1.005	1.005	**1.052 ***	**1.044**
		**6**	0.993	0.998	1.013	0.995	1.055	**1.058 ***	1.002	0.999	1.003	1.003	**1.051 ***	**1.048**	1.013	1.008	1.013	0.992	**1.064 ***	**1.064 ***	0.994	1.002	0.997	1.005	1.044	**1.05 ***
		**9**	1.005	0.998	1.0	0.987	**1.054 ***	**1.052**	0.999	1.001	0.999	1.003	**1.049 ***	**1.047**	1.005	1.006	1.007	1.01	**1.055 ***	**1.054**	0.997	0.998	1.0	0.996	1.047	**1.05 ***
		**12**	1.01	0.997	0.995	0.998	**1.059 ***	**1.029**	1.0	1.005	1.004	0.997	**1.05 ***	**1.049**	1.001	1.008	1.006	1.004	1.051	**1.053 ***	0.997	0.998	0.993	0.997	1.047	**1.05 ***
	**100**	**1**	1.009	0.988	0.994	0.999	**1.056 ***	**1.031**	0.999	0.998	1.007	0.999	1.049	**1.05 ***	1.005	1.004	1.009	1.004	**1.055 ***	**1.051**	0.998	0.997	0.997	0.996	1.047	**1.05 ***
		**3**	1.006	1.007	1.006	1.005	1.052	**1.055 ***	0.998	0.999	0.998	0.998	**1.048 ***	**1.048 ***	0.997	0.999	0.997	0.998	**1.047 ***	**1.047 ***	0.998	0.998	0.998	0.998	**1.048 ***	**1.048 ***
		**6**	1.006	1.005	1.006	1.002	**1.054 ***	**1.052**	0.998	0.998	0.997	1.0	**1.046 ***	**1.046 ***	0.999	0.997	0.998	1.0	**1.05 ***	**1.047**	0.998	0.998	0.998	1.0	**1.048 ***	**1.046**
		**9**	1.004	1.002	1.008	1.003	1.05	**1.051 ***	0.996	0.998	0.997	1.0	**1.047 ***	**1.044**	0.999	0.997	0.998	1.0	**1.053 ***	**1.045**	0.998	0.998	0.998	1.0	**1.049 ***	**1.047**
		**12**	1.002	1.005	1.007	1.004	**1.05 ***	**1.049**	0.997	0.999	0.999	0.998	**1.047 ***	**1.044**	1.001	0.998	0.998	1.001	**1.051 ***	**1.044**	0.997	0.999	0.998	1.0	**1.048 ***	**1.045**
	**1000**	**1**	0.999	1.0	1.0	1.0	**1.048 ***	**1.047**	0.996	0.998	0.998	1.0	**1.047 ***	**1.043**	1.0	0.997	0.998	1.001	**1.05 ***	**1.044**	0.998	0.999	1.0	1.0	**1.049 ***	**1.044**
		**3**	0.979	0.979	0.978	0.979	**1.028 ***	**1.027**	0.983	0.983	0.982	0.983	**1.032 ***	**1.031**	0.981	0.979	0.979	0.979	**1.03 ***	**1.028**	0.984	0.982	0.982	0.982	**1.034 ***	**1.031**
		**6**	0.979	0.979	0.979	0.982	**1.03 ***	**1.027**	0.983	0.983	0.983	0.987	**1.032 ***	**1.031**	0.98	0.979	0.979	0.981	**1.031 ***	**1.028**	0.985	0.982	0.985	0.986	**1.034 ***	**1.031**
		**9**	0.979	0.979	0.981	0.984	**1.03 ***	**1.027**	0.983	0.983	0.986	0.99	**1.032 ***	**1.031**	0.982	0.979	0.983	0.984	**1.03 ***	**1.027**	0.985	0.982	0.986	0.989	**1.034 ***	**1.031**
		**12**	0.98	0.979	0.982	0.987	**1.03 ***	**1.027**	0.985	0.984	0.986	0.991	**1.033 ***	**1.031**	0.982	0.98	0.986	0.986	**1.03 ***	**1.027**	0.986	0.984	0.988	0.99	**1.034 ***	**1.031**
**withmeds**	**10**	**1**	0.981	0.983	0.983	0.987	**1.03 ***	**1.027**	0.985	0.987	0.988	0.991	**1.034 ***	**1.031**	0.983	0.983	0.986	0.988	**1.031 ***	**1.027**	0.986	0.985	0.989	0.99	**1.034 ***	**1.031**
		**3**	0.047	0.118	0.075	**0.771 ***	0.086	**0.217**	0.0	1538.462	0.0	5025.804	**5250.0 ***	**0.0**	0.146	0.1	0.0	**0.691 ***	0.153	**0.153**	0.0	0.874	0.0	**1.428 ***	0.0	**0.0**
		**6**	0.412	0.416	0.311	**0.66 ***	0.464	**0.329**	1250.068	1538.526	0.036	**5025.726 ***	4200.12	**0.043**	0.212	0.239	0.127	**0.68 ***	0.223	**0.152**	0.673	0.968	0.384	**1.23 ***	0.707	**0.033**
		**9**	0.548	0.663	0.629	0.641	**0.721 ***	**0.425**	2222.561	1538.792	0.288	**5518.314 ***	5250.114	**2000.229**	0.203	0.392	0.486	**0.695 ***	0.213	**0.213**	0.47	**1.303 ***	0.733	1.099	0.493	**0.184**
		**12**	0.794	**0.807 ***	0.737	0.545	0.794	**0.591**	**6316.189 ***	1538.95	0.606	2365.371	6176.933	**4667.273**	0.539	0.569	0.631	**0.699 ***	0.566	**0.578**	0.807	**1.578 ***	0.598	1.136	0.739	**0.448**
	**100**	**1**	**0.88 ***	0.706	0.628	0.595	0.861	**0.678**	3750.445	1539.265	0.593	2365.322	**6300.474 ***	**0.628**	0.507	**0.793 ***	0.71	0.642	0.528	**0.503**	1.186	**1.635 ***	1.043	1.019	1.065	**0.72**
		**3**	0.033	0.062	0.106	**0.486 ***	0.067	**0.0**	0.0	0.075	0.139	**0.532 ***	0.0	**0.0**	0.0	0.152	0.016	**0.528 ***	0.0	**0.0**	0.0	0.039	0.06	**0.526 ***	0.0	**0.0**
		**6**	0.187	0.089	0.233	**0.498 ***	0.209	**0.122**	0.216	0.075	0.35	**0.541 ***	0.066	**0.197**	0.217	0.187	0.016	**0.561 ***	0.107	**0.098**	0.233	0.101	0.22	**0.531 ***	0.2	**0.099**
		**9**	0.321	0.228	0.445	**0.511 ***	0.348	**0.112**	0.298	0.124	0.447	**0.532 ***	0.191	**0.197**	0.218	0.275	0.186	**0.525 ***	0.154	**0.093**	0.363	0.235	0.342	**0.524 ***	0.348	**0.099**
		**12**	0.316	0.254	**0.578 ***	0.519	0.537	**0.426**	0.369	0.232	0.486	**0.517 ***	0.438	**0.497**	0.502	0.435	0.381	**0.565 ***	0.481	**0.509**	0.439	0.278	0.428	**0.547 ***	0.444	**0.411**
	**1000**	**1**	0.354	0.357	**0.618 ***	0.538	0.598	**0.579**	0.42	0.414	0.531	0.577	0.538	**0.598 ***	0.561	0.556	0.47	0.591	**0.625 ***	**0.541**	0.493	0.436	0.506	0.527	**0.559 ***	**0.461**
		**3**	0.0	0.035	0.0	**0.43 ***	0.0	**0.0**	0.084	0.0	0.039	**0.458 ***	0.0	**0.0**	0.0	0.0	0.095	**0.508 ***	0.0	**0.0**	0.0	0.046	0.225	**0.458 ***	0.0	**0.0**
		**6**	0.181	0.081	0.238	**0.472 ***	0.247	**0.27**	0.217	0.204	0.188	**0.507 ***	0.06	**0.172**	0.242	0.119	0.16	**0.514 ***	0.299	**0.225**	0.106	0.198	0.215	**0.476 ***	0.142	**0.137**
		**9**	0.338	0.26	0.388	**0.457 ***	0.375	**0.259**	0.421	0.363	0.248	**0.482 ***	0.193	**0.203**	0.285	0.291	0.33	**0.517 ***	0.349	**0.216**	0.391	0.364	0.283	**0.462 ***	0.259	**0.137**
		**12**	0.564	0.288	0.531	0.503	**0.681 ***	**0.587**	0.482	0.405	0.498	0.504	0.46	**0.555 ***	0.508	0.404	**0.573 ***	0.517	0.47	**0.457**	0.527	0.516	0.344	0.498	**0.537 ***	**0.442**
**sklearn_smal -red**	**10**	**1**	0.52	0.436	0.605	0.496	**0.63 ***	**0.607**	0.544	0.45	**0.646 ***	0.527	0.537	**0.61**	0.562	0.466	**0.647 ***	0.562	0.567	**0.51**	0.612	0.696	0.416	0.534	**0.705 ***	**0.516**
		**3**	**0.8 ***	0.784	0.79	0.743	0.799	**0.703**	0.77	0.8	0.862	0.846	**0.877 ***	**0.642**	0.382	**0.689 ***	0.598	0.561	0.382	**0.422**	0.574	**0.883 ***	0.737	0.781	0.575	**0.495**
		**6**	0.763	0.817	**0.931 ***	0.817	0.773	**0.682**	0.885	0.897	0.894	**0.933 ***	0.907	**0.682**	0.612	0.824	0.934	**0.942 ***	0.612	**0.624**	0.76	0.922	0.938	**0.961 ***	0.76	**0.728**
		**9**	0.865	0.836	**0.962 ***	0.864	0.935	**0.796**	0.922	0.941	**0.988 ***	0.954	0.937	**0.788**	0.884	0.976	0.961	**0.999 ***	0.884	**0.843**	0.799	0.905	**1.013 ***	0.947	0.851	**0.777**
		**12**	0.898	0.869	**0.964 ***	0.942	0.927	**0.909**	0.937	0.939	0.936	0.959	**0.968 ***	**0.928**	0.884	**1.025 ***	0.95	1.001	0.884	**0.895**	0.863	0.955	0.996	**1.016 ***	0.919	**0.947**
	**100**	**1**	0.932	0.871	0.978	0.985	**0.99 ***	**0.915**	0.95	**0.995 ***	0.93	0.97	0.984	**0.939**	0.932	0.965	0.975	**1.047 ***	0.901	**0.956**	0.968	0.965	1.023	**1.032 ***	0.994	**0.94**
		**3**	**0.747 ***	0.7	0.675	0.709	0.704	**0.635**	0.698	0.673	0.725	**0.729 ***	0.7	**0.65**	0.628	0.552	**0.724 ***	0.603	0.628	**0.531**	0.722	0.654	**0.74 ***	0.702	0.721	**0.618**
		**6**	**0.84 ***	0.769	**0.84 ***	0.775	0.784	**0.603**	0.813	0.75	0.815	0.842	**0.862 ***	**0.643**	0.803	0.767	**0.826 ***	0.81	0.822	**0.589**	0.761	0.802	**0.861 ***	0.806	0.763	**0.609**
		**9**	0.875	0.871	**0.928 ***	0.904	0.889	**0.731**	0.917	0.836	0.893	0.917	**0.933 ***	**0.757**	0.848	**0.885 ***	0.882	0.877	0.864	**0.722**	0.845	0.871	**0.897 ***	0.877	0.844	**0.744**
		**12**	0.917	**0.954 ***	0.94	**0.954 ***	0.918	**0.939**	0.954	0.927	0.904	0.95	**0.962 ***	**0.943**	0.883	0.918	0.934	**0.936 ***	0.886	**0.931**	0.881	**0.964 ***	0.934	0.926	0.878	**0.933**
	**1000**	**1**	0.954	0.99	0.985	0.984	0.958	**1.021 ***	0.974	0.969	0.939	**0.996 ***	0.978	**0.969**	0.917	0.945	0.966	0.961	0.921	**0.967 ***	0.94	**0.979 ***	0.978	0.956	0.943	**0.978**
		**3**	0.565	0.596	**0.694 ***	0.663	0.565	**0.545**	0.679	0.599	**0.696 ***	0.689	0.672	**0.556**	0.659	0.666	0.676	**0.701 ***	0.659	**0.575**	0.63	0.621	**0.722 ***	0.57	0.63	**0.569**
		**6**	0.739	0.77	0.775	**0.792 ***	0.739	**0.553**	0.714	0.774	0.794	**0.8 ***	0.706	**0.58**	0.788	0.756	**0.819 ***	0.772	0.788	**0.574**	0.789	0.728	**0.802 ***	0.753	0.783	**0.575**
		**9**	0.869	0.854	**0.872 ***	0.866	0.849	**0.726**	0.86	0.85	0.847	0.845	**0.866 ***	**0.727**	0.856	0.849	0.861	0.825	**0.866 ***	**0.72**	**0.878 ***	0.839	**0.878 ***	0.812	0.871	**0.728**
		**12**	0.912	**0.934 ***	0.928	0.933	0.895	**0.916**	0.927	0.906	0.876	0.907	**0.928 ***	**0.914**	0.871	**0.933 ***	0.889	0.9	0.874	**0.916**	0.891	**0.91 ***	0.904	0.873	0.879	**0.91 ***
**sklearn_large**	**10**	**1**	0.954	0.963	0.969	0.956	0.957	**0.995 ***	0.966	0.94	0.946	0.939	0.965	**0.994 ***	0.919	0.956	0.972	0.947	0.924	**0.993 ***	0.934	0.943	0.949	0.926	0.928	**0.991 ***
		**3**	0.857	**0.909 ***	0.881	0.87	0.856	**0.827**	**0.828 ***	0.802	0.774	0.813	0.784	**0.811**	0.496	0.713	0.388	**0.951 ***	0.496	**0.486**	0.561	0.661	0.835	0.729	**0.888 ***	**0.825**
		**6**	0.861	0.912	0.845	0.888	0.873	**0.943 ***	0.814	0.859	0.816	**0.872 ***	0.747	**0.854**	0.88	**1.138 ***	0.887	1.043	0.88	**0.893**	0.91	**1.06 ***	0.982	0.849	0.889	**0.922**
		**9**	0.908	0.886	0.837	0.898	0.947	**0.97 ***	0.854	0.843	0.819	0.806	0.82	**0.919 ***	1.294	1.161	1.11	1.133	1.297	**1.322 ***	0.908	**1.033 ***	1.006	0.951	0.914	**0.922**
		**12**	0.897	0.92	0.945	**0.949 ***	0.907	**0.903**	0.894	0.796	0.885	0.82	0.817	**0.913 ***	1.292	1.155	1.231	1.053	1.292	**1.307 ***	0.901	1.034	**1.058 ***	0.933	0.907	**0.909**
	**100**	**1**	0.889	0.948	**0.974 ***	0.941	0.915	**0.954**	0.907	0.798	0.873	0.837	0.826	**0.963 ***	1.382	1.12	1.23	1.1	1.382	**1.383 ***	0.937	**1.085 ***	1.011	0.94	0.93	**1.014**
		**3**	0.738	**0.808 ***	0.78	0.748	0.77	**0.722**	0.678	0.692	**0.753 ***	0.684	0.708	**0.682**	0.681	0.51	0.621	**0.704 ***	0.681	**0.621**	0.707	0.65	**0.756 ***	0.748	0.707	**0.675**
		**6**	0.827	0.863	0.832	0.781	0.796	**0.867 ***	0.772	0.754	**0.823 ***	0.716	0.804	**0.812**	0.748	0.766	**0.84 ***	0.795	0.749	**0.822**	0.746	0.834	0.801	0.804	0.744	**0.851 ***
		**9**	0.864	0.89	0.888	0.865	0.815	**0.971 ***	0.818	0.772	0.843	0.768	0.842	**0.934 ***	0.863	0.835	0.874	0.912	0.857	**0.929 ***	0.849	0.855	0.82	0.879	0.849	**0.942 ***
		**12**	0.928	0.881	0.879	0.895	0.944	**0.963 ***	0.866	0.85	0.891	0.844	0.891	**0.92 ***	0.882	0.873	0.914	**0.976 ***	0.88	**0.92**	0.907	0.86	0.852	0.912	0.894	**0.921 ***
	**1000**	**1**	0.959	0.887	0.933	0.936	0.964	**1.072 ***	0.895	0.852	0.908	0.885	0.901	**1.013 ***	0.94	0.911	0.913	0.988	0.931	**1.025 ***	0.934	0.885	0.878	0.942	0.923	**1.023 ***
		**3**	0.609	0.682	0.66	**0.688 ***	0.603	**0.624**	0.668	**0.679 ***	0.649	0.642	0.641	**0.574**	0.628	0.617	0.598	0.597	**0.642 ***	**0.486**	0.613	**0.659 ***	0.617	0.646	0.626	**0.568**
		**6**	0.702	0.663	0.737	0.682	0.689	**0.741 ***	0.737	0.67	**0.765 ***	0.684	0.762	**0.746**	0.641	0.671	0.738	0.683	0.645	**0.74 ***	0.647	0.652	**0.748 ***	0.668	0.657	**0.738**
		**9**	0.774	0.689	0.784	0.759	0.768	**0.847 ***	0.788	0.687	0.812	0.753	0.801	**0.856 ***	0.668	0.679	0.827	0.81	0.683	**0.843 ***	0.688	0.671	0.817	0.764	0.692	**0.831 ***
		**12**	0.8	0.744	0.804	0.793	0.771	**0.845 ***	0.839	0.749	0.819	0.773	**0.882 ***	**0.856**	0.74	0.769	**0.848 ***	0.835	0.754	**0.841**	0.733	0.76	**0.852 ***	0.824	0.734	**0.832**
